# Assessing external exposome by implementing an Environmental Data Management System using Open Data

**DOI:** 10.1038/s41598-024-62924-0

**Published:** 2024-07-26

**Authors:** Sofia Tagliaferro, Sara Maio, Federico Pirona, Ilaria Stanisci, Giuseppe Sarno, Patrizia Silvi, Marianthi Kermenidou, Nafsika Papaioannou, Reena Perchard, Igor Prpic, Kinga Polanska, Joanna Jerzynska, Elisabete Ramos, Joaquim Rovira, Jordina Belmonte, Janja Snoj Snoj Tratnik, Milena Horvat, David Kocman, Zdravko Spiric, Jacqueline Zickella, Salvatore Fasola, Stefania La Grutta, Velia Malizia, Laura Montalbano, EarlyFOOD, Bart Keijser, Jasper Kieboom, Martin Larsen, Marta Schumacher, Tim van den Broek, Rémy Villette, HEALS EXHES, Nour Baiz, Henrique Barros, John Bartzis, Norhidayah Binti Ahmad, Beatrice Bocca, Sonia Brescianini, Gemma Calamandrei, Anthoula Chatzimpaloglou, Eugenia Dogliotti, Ingrid Falnoga, Maria João Fonseca, Catherine Gabriel, Amir Gamil, Alberto Gotti, Wojciech Hanke, Edward Johnstone, Joanna Jurewicz, Michael Kabesch, Katarzyna Kalska-Sochacka, Spyros Karakitsios, David Kocman, Vikas Kumar, Darja Mazej, Filomena Mazzei, Ettore Meccia, Luisa Minghetti, Lorenza Nisticò, Eduardo de Oliveira Fernandes, Reena Perchard, Anna Pino, Dimosthenis Sarigiannis, Marta Schumacher, Maria Antonietta Stazi, Kamila Szcześniak, Patrizia Tancredi, Gabriela Ventura Silva, Giovanni Viegi, Sandra Baldacci, Isabella Annesi-Maesano

**Affiliations:** 1 Pulmonary Environmental Epidemiology Unit, National Research Council (CNR), Institute of Clinical Physiology (IFC), Pisa, Italy; 2 Unit of Epidemiology and Medical Statistics, Department of Diagnostics and Public Health, University of Verona, Verona, Italy; 3 Environmental Engineering Laboratory, Department of Chemical Engineering, Aristotle University of Thessaloniki, Thessaloniki, Greece; 4 HERACLES Research Center On the Exposome and Health, Center for Interdisciplinary Research and Innovation, Aristotle University of Thessaloniki, Thessaloniki, Greece; 5 Division of Developmental Biology and Medicine, Faculty of Biology, Medicine and Health, Manchester Academic Health Science Centre, University of Manchester, Manchester, UK; 6 Department of Pediatrics, University of Rijeka, Clinical Hospital Centre Rijeka, Rijeka, Croatia; 7 Department of Environmental and Occupational Health Hazards, Nofer Institute of Occupational Medicine (NIOM), Lodz, Poland; 8 Department of Paediatrics and Allergy, Copernicus Memorial Hospital, Medical University of Łódź (MUL), Lodz, Poland; 9 Laboratório Para a Investigação Integrativa E Translacionalem Saúde Populacional (ITR), Porto, Portugal; 10 Departamento de Ciências da Saúde Pública E Forenses E Educação Médica, Faculdade de Medicina, Porto, Portugal; 11 EPIUnit, Instituto de Saúde Pública, Universidade Do Porto, Porto, Portugal; 12 Environmental Engineering Laboratory, Departament d’Enginyeria Quimica, Universitat Rovira I Virgili, Tarragona, Catalonia Spain; 13 Laboratory of Toxicology and Environmental Health, School of Medicine, IISPV, Universitat Rovira I Virgili, Reus, Catalonia Spain; 14 Institute of Environmental Science and Technology, Autonomous University of Barcelona, Bellaterra (Cerdanyola del Vallès), Barcelona, Catalonia Spain; 15 Department of Animal Biology, Plant Biology and Ecology, Autonomous University of Barcelona, Bellaterra (Cerdanyola del Vallès), Barcelona, Catalonia Spain; 16 Department of Environmental Sciences, Jožef Stefan Institute, Ljubljana, Slovenia; 17 Green Infrastructure Ltd, Zagreb, Croatia; 18 Dept of Pneumology, Allergology and Thoracic Oncology, CHU, Montpellier, France; 19 IDESP INSERM and Univ Mont, Montpellier, France; 20 National Research Council (CNR), Institute of Translational Pharmacology (IFT), Palermo, Italy; 21 Institute for Research and Innovation in Biomedicine (IRIB), National Research Council (CNR), Palermo, Italy; 22 Netherlands Organisation for Applied Scientific Research (TNO), Leiden, The Netherlands; 23 Sorbonne Université, INSERM U1135, Centre d’Immunologie Et Des Maladies Infectieuses (CIMI-Paris), Paris, France; 24 Environmental Engineering Laboratory, Universitat Rovira I Virgili, Tarragona, Spain; 25 Institute of Public Health, University of Porto, Porto, Portugal; 26 Department of Mechanical Engineering, University of Western Macedonia (UOWM), Sialvera and Bakola, Kozani, Greece; 27 Centre for Epidemiology, Division of Population Health, Health Services Research and Primary Care School of Health Sciences, Faculty of Biology, Medicine and Health, University of Manchester, Manchester, UK; 28 Italian National Institute of Health, Rome, Italy; 29 Faculty of Chemistry, Aristotle University of Thessaloniki, Thessaloniki, Greece; 30 School of Engineering, Aristotle University of Thessaloniki, Thessaloniki, Greece; 31 University Clinic Regensburg, Regensburg, Germany; 32 Institute of Mechanical Engineering, University of Porto, Porto, Portugal

**Keywords:** Air quality, Lifestyle, Noise, Pollen, Pesticides, Water quality, Environmental impact, Ecological epidemiology, Environmental sciences, Risk factors

## Abstract

Due to the increasing importance of exposome in environmental epidemiology, feasibility and usefulness of an Environmental Data Management System (EDMS) using Open Data was evaluated. The EDMS includes data from 10 European cities (Celje (Slovenia), Łódź (Poland), Manchester (UK), Palermo (Italy), Paris (France), Porto (Portugal), Regensburg (Germany), Reus (Spain), Rijeka (Croatia), Thessaloniki (Greece)) about external non-specific and specific exposome factors at the city or country level (2017–2020). Findings showed that the highest values of life expectancy were in Reus females (86 years) and Palermo males (81 years). UK had the highest obesity rate (28%), Croatia the highest prescribed drug consumption (62%), Greece and Portugal the highest smoking rates (37%, 42%) and daily alcohol consumption (21%), respectively. The most polluted cities were Thessaloniki for PM_10_ (38 µg/m^3^), Łódź for PM_2.5_ (25 µg/m^3^), Porto for NO_2_ (62 µg/m^3^) and Rijeka for O_3_ (92 µg/m^3^). Thessaloniki had the highest grey space (98%) and Łódź the highest cumulative amount of pollen (39,041 p/m^3^). The highest daily noise levels ≥ 55 dB was in Reus (81% to traffic) and Regensburg (21% to railway). In drinking water, arsenic had the highest value in Thessaloniki (6.4 µg/L), boron in Celje (24 mg/L) and lead in Paris (46.7 µg/L). Portugal and Greece showed the highest pesticide residues in food (7%). In conclusion, utilizing open-access databases enables the translation of research findings into actionable strategies for public health interventions.

## Introduction

To date, most epidemiological studies have focused on the effects of single environmental exposures, without considering the role of real-life multiple exposures. A major innovation has been provided by the introduction of the concept of the exposome, defined as the totality of exposures of an individual in a lifetime, and its health effects^1^. It consists of three domains: the external specific exposome, the external non-specific exposome and the internal exposome^1,2^. The external specific exposome consists of the exposures to a set of external environmental factors (e.g. air, soil and water chemicals, biological and physical pollutants, radiations, noise, etc.) to which individuals are personally exposed over lifetime^2^. The external non-specific exposome consists of generic exposures (e.g. climate, greenness, socioeconomic factors, etc.) usually assessed at the community level (neighbourhood, city, social group, etc.)^2^. The internal exposome consists of the internal body chemical environment, namely of signals resulting from internal processes (metabolic, inflammatory, omics, etc.) as a consequence of external environmental aggressions^2^.

Since Wild introduced the exposome concept, an increasing number of studies have applied it in the case of various diseases^1–6^. They have clearly indicated that as lifetime environmental exposure assessments are difficult to achieve in practice, the external exposome has to be estimated at different life stages^1,5,7^. In particular, the first years from conception onwards (1000 days of life) are considered a critical window, due to the physiological changes and metabolic development in fetal and postnatal life, often resulting in impaired health conditions like allergic/asthma and neurodevelopmental outcomes throughout the life span^4,5,7^.

As a whole, exposome studies have shown that environmental data may often be incomplete and are rarely standardized, despite the fact that the European Union has been moving toward the digitalization and the implementation of thematic harmonized datasets^8,9^. Furthermore, studies have also been affected by the lack of routine and Open Access FAIR data i.e. providing freely available, usable and sharable data on the considered risk factors^10^.

The HEALS (Health and Environment—wide Associations based on Large population Surveys, https://cordis.europa.eu/project/id/603946) and EarlyFOOD (Long-term impact of gestational and early-life dietary habits on infant gut immunity and disease risk, https://immulab.fr/cms/index.php/projects/earlyfood) were EU funded projects, which aim was to build and estimate the children’s internal and external exposomes to gain mechanistic insights into the development of major childhood diseases such as asthma, allergies, obesity, diabetes and neurodevelopmental troubles. In both projects, birth cohort data were used to implement exposome-health association studies (Environment Wide Association Studies—EnvWAS) and suggest prevention measures.

Within these two projects, an Environmental Data Management System (EDMS) was implemented, containing as much exposome data as possible freely available on the web. The EDMS is intended as a useful tool to support the scientific community and stakeholders dealing with environmental issues, as well as environmental policies.

The aim of this paper is to describe the methodology, usefulness and feasibility of EDMS implementation with Open-Access data on exposure to external non-specific and specific exposome from 10 European countries, in the context of the European HEALS/EarlyFOOD projects.

## Materials and methods

### Study design

An observational cross-sectional study has been undertaken to further implement EDMS with data on exposure to external non-specific and specific exposome.

#### Study cities/countries

**Figure 1:**
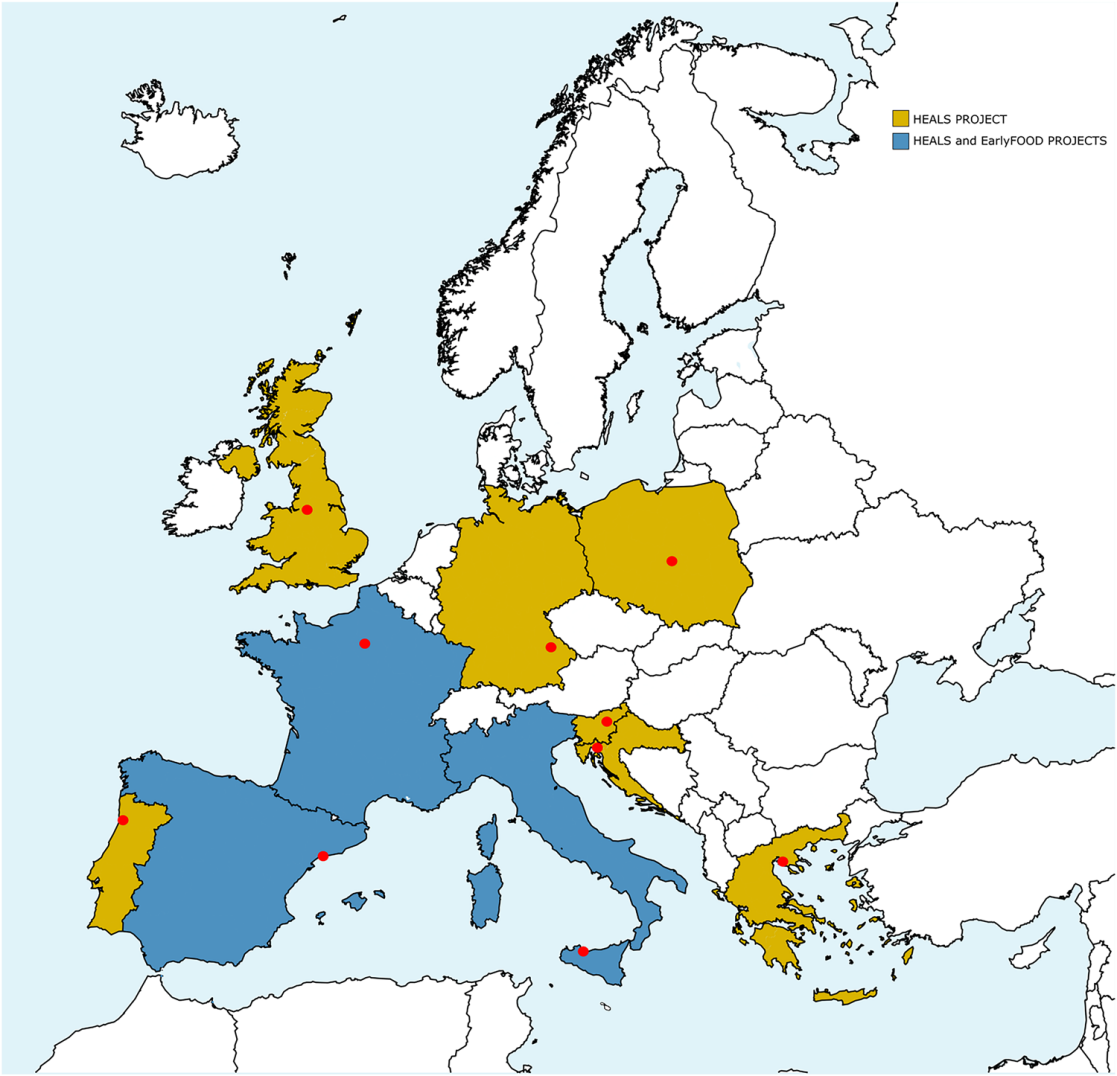
Map of the cities participating in the HEALS and EarlyFOOD projects (modified by MapChart, https://mapchart.net).

The HEALS project (2013–2019) integrated a comprehensive array of novel technologies, data analysis and modelling tools for supporting exposome-health association studies in a European birth cohort from ten European cities initiated in the frame of the Exposure and Health Examination Survey (EXHES): Celje (Slovenia), Łódź (Poland), Manchester (United Kingdom, UK), Palermo (Italy), Paris (France), Porto (Portugal), Regensburg (Germany), Reus (Spain), Rijeka (Croatia), Thessaloniki (Greece). These cities were chosen to represent different European meteo-climatic, geographical, geological, cultural, and behavioural characteristics (Fig. 1).

The EarlyFOOD project (2018–2022) investigated the external and internal exposome (metabolomics and microbiomics) for better understanding the development of asthma and allergies, obesity, diabetes and neurodevelopmental disorders. It involved birth cohorts from Palermo, Paris, and Reus, also participating in the HEALS study (Fig. 1).

Within the HEALS project, an EDMS, gathering information on environmental stressors (e.g. environmental pollution, food, bio-contaminants) in the ten cities participating in EXHES, was developed. Furthermore, within EarlyFOOD project, the EDMS was enriched with lifestyle, additional environmental stressors, and socio-economic characteristics for 2017–2020.

Due to the SARS-CoV-2 pandemic restrictions, the year 2020 is an outlier in relation to the considered stressors, thus it was decided to analyse it separately, wherever possible.

### External exposome factors and data collection

External non-specific exposome factors included socio-demographic characteristics (population, mortality rate, life expectancy at birth, population at risk of poverty), lifestyle factors (Body Mass Index (BMI), smoking habits, alcohol and drug consumption), climatic parameters (temperature and humidity), land use/land cover (LU/LC). External specific exposome included air pollution (PM_10_: Particulate Matter (PM) with an aerodynamic diameter smaller than 10 µm; PM_2.5_: PM with an aerodynamic diameter smaller than 2.5 µm; NO_2_: Nitrogen Dioxide; O_3_: Ozone), pollen/spores, noise (from traffic and railway), drinkable water, and pesticide residues in food. In order to implement the EDMS, we conducted thorough research on supplier websites at European, national, and local levels to collect relevant data concerning the factors mentioned above. In cases where Open Data were unavailable, data were requested from the respective institutions to complete the EDMS. The current paper deals with socio-economic, lifestyle and environmental data at city or country level in accordance to the relative copyright policy (Table S4). For each factor, all the details on considered period and metrics are reported in the Supplementary Information (Tables S1—S4: data source, considered variables, temporal resolution, provided statistics, data availability and inter-cities comparability). Briefly, whenever feasible, we downloaded data at the city level from single European databases, ensuring consistency in temporal resolution to facilitate uniformity of information. Basic statistical analyses were performed to allow data comparability, such as means over the specified periods, particularly in cases where data did not entirely cover the 2017–2020 period.

No experiments on humans and/or use of human tissue samples occurred in this study as well as no use of personal data (e.g. health, location, lifestyle, job).

## Results

### External non-specific exposome

#### Socio-demographic characteristics

Table 1 shows socio-demographic characteristics stratified by city. Paris was the city with the highest total mean population (n = 2,168,614), and overall females overcome males (average across cities: 52% vs. 48%). In 2017–2019, the highest mortality rate was registered in Łódź, Porto and Rijeka (14%) and the highest mean percentage of the population at risk of poverty was in Spain (21%). In 2020, a general increase in the mortality rate was shown while risk of poverty slightly increased only in France (+ 1%).

**Table 1:** External non-specific exposome factors between 2017 and 2020 in the ten cities contributing to HEALS and EarlyFOOD projects.

		Celje (Slovenia)	Łódź (Poland)	Manchester (UK)	Palermo (Italy)	Paris (France)	Porto (Portugal)	Regensburg (Germany)	Reus (Spain)	Rijeka (Croatia)	Thessaloniki (Greece)
**Socio-demographic indicators**											
Total population (n)	2017–2020	37,946	688,038	550,432	653,563	2,168,614	215,149	151,309	104,285	118,357	1105615^a^
Female population (%)	2017–2020	50	54	49	52	53	55	52	52	53	53^a^
Male population (%)	2017–2020	50	46	51	48	47	45	48	48	47	47^a^
Total mortality rate (%)	2017–2019	9^b^	14	6	10	6	14	NA	9	14	10^a^
	2020	11^b^	16	8	11	8	15	NA	10	16	12^a^
Population at risk of poverty (%)*	2017–2019	13	15	18	20	13	18	16	21	19	19
	2020	12	15	NA	20	14	16	16	21	18	18
Life expectancy at birth (mean age)*:											
female	2017–2020	84	81	83^c^	85	85^d^	84	83^c^	86	81	83
male	2017–2020	78	74	79^c^	81	80^d^	78	79^c^	80	75	79
Lifestyle risk factors *											
Smoking prevalence (%)	2017	28	30	17	25	36	26	25	27	35	37
	2020	27	26	12	23	28	21	23	24	36	42
BMI (%):											
< 18.5 (underweight)	2019	2	3	2	4	4	2	3	2	12	1
≥ 18.5 and < 25 (normalweight)	2019	42	41	34	51	50	43	45	45	35	42
≥ 25 and < 30 (pre-obese)	2019	37	38	36	33	31	37	34	37	41	40
≥ 30 (obese)	2019	19	19	28	11	14	17	19	15	23	16
Alcohol consumption (%):											
every day	2019	7	2	NA	12	10	21	8	13	10	6
every week	2019	24	17	NA	29	34	22	32	23	18	25
every month	2019	25	30	NA	16	23	15	26	18	21	28
less than once a month	2019	18	26	NA	8	10	12	13	13	13	13
never	2019	26	26	NA	35	23	30	21	33	38	29
Drugs consumption (in the last two weeks) (%):											
prescribed	2019	48	49	NA	38	49	56	54	51	62	44
not prescribed	2019	34	45	NA	19	31	23	37	15	38	20
**Climatic parameters**											
Temperature (°C)	2017–2020	11	9	10	19	13	NR	10	17	15	18
Relative humidity (%)	2017–2020	77	NR	82	61	73	NA	76	68	63	NA
**Land use/land cover**											
Agricultural (% of area)	2018	NA	33	32	11	3	21	35	69	5	0
Blue (% of area)	2018	NA	0	5	0	1	3	2	0	1	1
Green (% of area)	2018	NA	17	16	32	16	31	8	3	84	1
Grey (% of area)	2018	NA	50	47	57	80	45	55	28	10	98

*Data at country level; NA: Not Available, data information not provided; NR: Not Reliable, data were available but insufficient data coverage; ^a^prefecture; ^b^province; ^c^computed over two years before and the current year; ^d^estimation for 2019–20 not definitive; BMI: Body Mass Index; °C: Celsius degree.

Life expectancy at birth was generally higher in Reus females (86 years) and Palermo males (81 years).

#### Lifestyle risk factors

Country lifestyle risk factors are reported in Table 1. In 2017 smoking prevalence was highest in Greece (37%), and a general decrease was shown in 2020, except for Greece and Croatia (+ 5% and + 1%, respectively).

In 2019, the highest percentage of pre-obese (25 ≤ BMI < 30 kg/m^2^) and obese (BMI ≥ 30 kg/m^2^) population resulted in Croatia (41%) and the UK (28%). As regards alcohol consumption, the highest “every day” and “every week” consumption was in Portugal (21%) and France (34%), respectively. In contrast, the highest percentage of “never” alcohol consumption was in Croatia (38%). On the other hand, drug consumption was highest in Croatia (62% prescribed) and Poland (45% not prescribed).

#### Climatic parameters

As expected according to geographical location, the hottest and wettest cities were Palermo (19 °C) and Manchester (82%), respectively (Table 1).

#### Land use/land cover

The highest percentage of the agricultural, blue, green, grey areas was in Reus (69%), Manchester (5%), Rijeka (84%), and Thessaloniki (98%), respectively (Table 1).

### External specific exposome

#### Air quality

In 2017–2019, the cities with the highest value of air pollutants (µg/m^3^) were: Thessaloniki for PM_10_ (38), Łódź for PM_2.5_ (25), Porto for NO_2_ (62), and Rijeka for summer O_3_ (92). In 2020 a general decrease in PM and NO_2_ values was shown in all cities with available data, while there was not a specific uniform trend for O_3_ (Table 2).

**Table 2:** External specific exposome factors between 2017 and 2020 in the ten cities contributing to HEALS and EarlyFOOD projects.

		Celje (Slovenia)	Łódź (Poland)	Manchester (UK)	Palermo (Italy)	Paris (France)	Porto (Portugal)	Regensburg (Germany)	Reus (Spain)	Rijeka (Croatia)	Thessaloniki (Greece)
**Air quality**											
PM_10_ (µg/m^3^)	2017–2019	*28*	*35*	*21*	*28*	*25*	NR	*19*	*18*	NR	*38*
	2020	*22*	*28*	15	*20*	*22*	NA	*18*	*19*	NA	*35*
PM_2.5_ (µg/m^3^)	2017–2019	NA	*25*	*10*	*11*	*15*	NR	NA	NA	*10*	*21*
	2020	*17*	*17*	*8*	*11*	*12*	NA	NA	NA	*9*	*19*
NO_2_ (µg/m^3^)	2017–2019	*26*	*23*	*40*	*37*	***45***	***62***	*38*	*18*	*13*	*26*
	2020	*20*	*23*	*26*	*15*	*30*	NA	*30*	*14*	*12*	*22*
O_3_ (µg/m^3^) (April-September)	2017–2019	60	66	41	78	55	42	NA	72	92	73
	2020	56	66	46	80	64	56	NA	65	NR	69
**Pollen and spores**											
*Pollen* (p/m^3^):											
Asteraceae:											
*Ambrosia*	2017–2020	649	197	3	30	11			NA	437	21
*Artemisia*	2017–2020	177	847	28	32	93			139	NA	23
Betulaceae:											
*Alnus*	2017–2020	2789	6612	132	18	2050			128	762	127
*Betula*	2017–2020	4943	14,918	1885	8	4112			65	1272	52
Corylaceae:											
*Carpinus*	2017–2020	NA	201	NA	6	482			NA	1698	637
*Corylus*	2017–2020	1714	480	100	11	858			434	603	118
Cupressaceae	2017–2020	5054	NA	NA	8518	4312			11,459	15,052	6727
Fagaceae:											
*Fagus*	2017–2020	2269	72	NA	31	55			2	1431	42
*Quercus*	2017–2020	2138	2041	426	296	1939			4469	4042	2941
Oleaceae:	2017–2020	NA	NA	NA	769	NA			5110	NA	295
*Olea*	2017–2020	1517	NA	NA	486	9			4638	437	250
Poaceae	2017–2020	3047	4156	4829	210	2524			744	556	165
Urticaceae	2017–2020	12,978	9517	3117	4986	5184			2355	7987	3741
cAPI	2017–2020	37,273	39,041	10,520	14,915	21,629			24,905	34,277	14,889
*Spores* (s/m^3^):											
*Alternaria*	2017–2020	NA	25,499	NA	2954	23,297			12,732	NA	3580
*Cladosporium*	2017–2020	NA	1,110,795	NA	NA	627,339			245,880	NA	60,514
**Noise**											
*Traffic noise* (% population exposed)											
Lden: ≥ 55 dB	2017	NA	34	35	50	78	16	21	81	21	NA
Lnight: ≥ 50 dB	2017	NA	38	24	37	54	12	12	59	13	NA
*Railway noise* (% population exposed)											
Lden: ≥ 55 dB	2017	NA	1	2	0	12	1	21	1	1	NA
Lnight: ≥ 50 dB	2017	NA	1	2	NA	10	1	16	0	0	NA
**Drinking water**											
1,2-dichloroethane (µg/L)	2017–2019	0.2	NA	NA	NA	< 0.5	< 0.75		NA	< 0.75	< 0.15
	2020	0.2	< 0.1	NA	NA	< 0.5	< 0.75		NA	NA	NA
Acrylamide (µg/L)	2017–2019	NA	NA	NA	NA	NA	< 0.05		NA	< 0.04	NA
	2020	NA	NA	NA	NA	NA	< 0.05		NA	NA	NA
Antimony (µg/L)	2017–2019	0.7	NA	NA	NA	< 1	< 1		NA	< 1.2	< 1
	2020	0.5	< 1	NA	NA	< 1	< 1		NA	NA	NA
Arsenic (µg/L)	2017–2019	0.5	NA	NA	NA	< 2	5.4		NA	< 0.4	6.4
	2020	1	< 1	NA	NA	< 2	3.3		NA	NA	NA
Benzene (µg/L)	2017–2019	0.3	NA	NA	NA	< 0.5	< 0.3		NA	< 0.4	< 0.05
	2020	0.3	< 0.1	NA	NA	< 0.5	< 0.2		NA	NA	NA
Benzo(a)pyrene (µg/L)	2017–2019	0.003	NA	NA	NA	< 0.005	< 0.02		NA	< 0.002	< 0.00135
	2020	0.002	< 0.002	NA	NA	0.0003	< 0.005		NA	NA	NA
Boron (mg/L)	2017–2019	0.03	NA	NA	NA	0.03	< 0.1		NA	< 0.05	0.21
	2020	***24***	< 0.1	NA	NA	0.03	0.01		NA	NA	NA
Bromate (µg/L)	2017–2019	NA	NA	4.8	NA	3.83	< 5		NA	< 2	6.9
	2020	2.5	NA	NA	NA	7.4	< 5		NA	NA	NA
Cadmium (µg/L)	2017–2019	0.05	NA	NA	< 1	< 1	< 1		< 0.5	0.03	< 0.5
	2020	0.05	< 0.3	NA	NA	< 1	< 0.2		< 0.5	NA	NA
Chlorates and chlorites (mg/L)	2017–2019	NA	NA	NA	NA	NA	NA		NA	NA	NA
	2020	NA	**0.436**	NA	NA	NA	NA		NA	NA	NA
Chromium (µg/L)	2017–2019	1.3	NA	NA	< 1	< 5	< 5		< 5	0.8	< 5
	2020	1	< 2	NA	NA	< 5	< 1		< 2.5	NA	NA
Copper (mg/L)	2017–2019	0.004	NA	0.06	NA	0.04	0.01		< 0.02	0.0008	0.02
	2020	2.1	< 0.004	0.06	NA	NA	0.15		< 0.05	NA	NA
Cyanide (µg/L)	2017–2019	NA	NA	NA	NA	< 10	< 10		NA	< 10	ND
	2020	5	< 20	NA	NA	0.24	< 10		NA	NA	NA
*Enterococci* (CFU/100 mL)	2017–2019	0	NA	NA	0	< 1	***1***		0	0	NA
	2020	0	0	NA	NA	< 1	***32***		0	NA	NA
Epichlorohydrin (µg/L)	2017–2019	NA	NA	NA	NA	NA	< 0.1		NA	< 0.03	NA
	2020	NA	NA	NA	NA	NA	< 0.1		NA	NA	NA
*Escherichia coli* (CFU/100 mL)	2017–2019	***0.3***	NA	NA	0	< 1	***6***		0	0	< 1
	2020	0	0	NA	NA	< 1	***2***		0	NA	NA
Fluoride (mg/L)	2017–2019	0.05	NA	0.7	0.35	0.21	0.12		0.27	0.04	0.15
	2020	0.1	< 0.14	0.7	NA	0.27	0.11		0.3	NA	NA
Haloacetic acids (HAA) (µg/L)	2017–2019	NA	NA	9.6	NA	NA	NA		NA	NA	NA
	2020	NA	NA	10	NA	NA	NA		NA	NA	NA
Lead (µg/L)	2017–2019	1.5	NA	< 1	< 1	***46.7***	< 3		< 3	< 0.3	**5.4**
	2020	0.5	< 2	1	NA	NA	< 1		< 1	NA	NA
Mercury (µg/L)	2017–2019	0.1	NA	NA	NA	< 0.5	< 0.3		NA	< 0.25	< 0.2
	2020	0.1	< 1	NA	NA	< 0.5	0.14		NA	NA	NA
Nickel (µg/L)	2017–2019	5	NA	NA	< 2	< 5	< 6		< 3	< 0.8	5
	2020	2	< 4	NA	NA	NA	< 2		< 2.5	NA	NA
Nitrate (mg/L)	2017–2019	44.1	NA	NA	8.3	45.8	5.7		12.9	4.4	10.1
	2020	46.9	< 2.2	NA	NA	47	6.2		12.9	NA	NA
Nitrite (mg/L)	2017–2019	0.01	NA	NA	< 0.05	< 0.02	0.02		< 0.05	< 0.5	< 0.05
	2020	0.005	< 0.04	NA	NA	< 0.02	0.11		< 0.03	NA	NA
Polycyclic aromatic hydrocarbons (PAHs) (µg/L)	2017–2019	0.01	NA	NA	NA	< 0.02	< 0.08		NA	< 0.01	< 0.1
	2020	0.005	< 0.004	NA	NA	0.001	< 0.08		NA	NA	NA
Selenium (µg/L)	2017–2019	0.5	NA	NA	NA	< 2	< 3		NA	< 0.5	< 1
	2020	1	< 1	NA	NA	< 2	8		NA	NA	NA
Tetrachloroethene and trichloroethene (µg/L)	2017–2019	1.3	NA	NA	NA	< 0.5	< 0.5		NA	< 0.1	< 0.5
	2020	0.9	< 0.1	NA	NA	NA	< 0.5		NA	NA	NA
Total pesticides (µg/L)	2017–2019	0.03	NA	NA	NA	0.09	< 0.1		NA	NA	< 0.5
	2020	0.03	< 0.02	NA	NA	0.09	< 0.1		NA	NA	NA
Total Trihalomethanes (TTHMs) (µg/L)	2017–2019	2.6	NA	10.3	NA	36.2	42.2		NA	NA	13.8
	2020	2.6	23.13	5.1	NA	56.3	32		NA	NA	NA
Vinyl chloride (µg/L)	2017–2019	NA	NA	NA	NA	< 0.5	< 0.1		NA	< 0.03	NA
	2020	NA	NA	NA	NA	< 0.5	< 0.1		NA	NA	NA
Food pesticides*											
Pesticides residues > MRLs (%)	2017–2019	1	5	4	2	4	7	2	3	2	5
	2020	4	6	4	2	5	6	4	4	6	7

*Data at country level; NA: Not Available, data information not provided; NR: Not Reliable, data were available but insufficient data coverage; ND: Not detected; PM_10_: particulate matter with an aerodynamic diameter smaller than 10 µm; PM_2.5_: particulate matter with an aerodynamic diameter smaller than 2.5 µm; NO_2_: Nitrogen Dioxide; O_3_: Ozone; µg/m^3^: microgram/cubic meter; p/m^3^: pollen/cubic meter; cAPI: cumulative Allergenic Pollen Integral; s/m^3^: spores/cubic meter; dB: decibel; Lden: day-evening-night-weighted sound pressure level average in a year; Lnight: night-weighted sound pressure level average in a year; mg/L: milligram/Liter; µg/L: microgram/Liter; CFU/100 mL: colony forming unit/100 milliliter; MRLs: Maximum Residue Levels; in *Italics* the value higher than the WHO guidelines; in **bold** the value higher than the EU limits; in ***Italics bold*** the value exceeding both WHO guidelines and EU limits. Empty cells indicate that the source site/database was not found.

#### Pollen and spores

Regarding pollen presence, Celje had the highest values (p/m^3^) of *Ambrosia* (649), *Corylus* (1714), *Fagus* (2269), Urticaceae (12,978); Łódź of *Artemisia* (847), *Alnus* (6612), *Betula* (14,918); Manchester of Poaceae (4829); Reus of *Quercus* (4469), Oleaceae (5110); Rijeka of *Carpinus* (1698), Cupressaceae (15,052). Overall, the total pollen load (cAPI) for the period 2017–2020, had the highest value in Łódź (39,041 p/m^3^). Instead, the highest value of spores’ concentration (s/m^3^) was in Łódź (*Alternaria*: 25,499; *Cladosporium*: 1,110,795) (Table 2).

#### Noise

Reus reported the highest percentage of high traffic noise levels exposure during the day and the night (Lden ≥ 55 dB: 81%; Lnight ≥ 50 dB: 59%), and Regensburg the highest percentage of high railway noise levels exposure (Lden ≥ 55 dB: 21%; Lnight ≥ 50 dB: 16%) (Table 2).

#### Drinking water

In 2017–2019, the cities with the highest mean values of one or more contaminants were: Thessaloniki (arsenic: 6.4 µg/L; boron: 0.21 mg/L; bromate: 6.9 µg/L), Celje (1,2-dichloroethane: 0.2 µg/L; antimony: 0.7 µg/L; benzene: 0.3 µg/L; benzo(a)pyrene: 0.003 µg/L; cadmium: 0.05 µg/L; chromium: 1.3 µg/L; mercury: 0.1 µg/L; polycyclic aromatic hydrocarbons (PAHs): 0.01 µg/L; selenium: 0.5 µg/L; tetrachloroethene and trichloroethene: 1.3 µg/L), Manchester (copper: 0.06 mg/L; fluoride: 0.7 mg/L), Paris (lead: 46.7 µg/L; nitrate: 45.8 mg/L; total pesticides: 0.09 µg/L), Porto (nitrite: 0.02 mg/L; Total Trihalomethanes (TTHMs): 42.2 µg/L; *Enterococci*: 1 CFU/100 mL; *Escherichia coli*: 6 CFU/100 mL). The values of nickel were highest in Celje and Thessaloniki (5 µg/L).

In 2020, the cities with the highest values were: Paris (bromate: 7.4 µg/L; nitrate: 47 mg/L; TTHMs: 56.3 µg/L; total pesticides: 0.09 µg/L), Celje (1,2-dichloroethane: 0.2 µg/L; antimony: 0.5 µg/L; benzene: 0.3 µg/L; benzo(a)pyrene: 0.002 µg/L; boron: 24 mg/L; cadmium: 0.05 µg/L; chromium: 1 µg/L; copper: 2.1 mg/L; cyanide: 5 µg/L; nickel: 2 µg/L; PAHs: 0.005 µg/L; tetrachloroethene and trichloroethene: 0.9 µg/L), Manchester (fluoride: 0.7 mg/L; lead: 1 µg/L), Porto (arsenic: 3.3 µg/L; mercury: 0.14 µg/L; nitrite: 0.11 mg/L; selenium: 8 µg/L; *Enterococci*: 32 CFU/100 mL; *Escherichia coli*: 2 CFU/100 mL) (Table 2).

#### Food pesticides

In 2017–2019, the highest percentage of food pesticide residues (Maximum Residue Levels (MRLs)) was observed in Portugal (7%), and a general increasing in the values was shown in 2020 (Table 2).

## Discussion

The exposome refers to the sum of all environmental exposures that an individual experiences throughout the lifetime. Building the exposome requires collecting and integrating data from a variety of sources, including personal monitoring devices, biomonitoring, and environmental monitoring.

This paper reports the accessibility and usability of Open Data on exposure to the external non-specific (socio-demographic and lifestyle risk factors, climatic parameters, LU/LC) and specific (air, water and noise pollution, pollen/spores, food pesticides) exposome in 10 European cities/countries participating in the HEALS and EarlyFOOD projects.

Through the EDMS, inter-cities differences in demographic and environmental aspects have emerged which could affect children’s development.

Socio-economic status and lifestyle are significant determinants of children’s health and wellbeing^11^. The results showed that Poland, UK and Croatia had lower life expectancies and higher rates of pre-obesity/obesity. It is well-known that a higher BMI in childhood and adolescence is associated with adverse health consequences throughout the lifespan^12^. Also, in Croatia and Poland, the higher consumption of drugs represented a potential higher morbidity risk for new generations. Children living in western-central Europe might be at higher poverty risk and be more susceptible to indirect consequences of high parental alcohol consumption, such as abandonment and violence^11^. Given the high smoking prevalence rates, France, Greece, and Croatia displayed a likely higher prevalence of Environmental Tobacco Smoking. Fetal exposure to tobacco and alcohol increases the risk of adverse pregnancy outcomes and other health problems in the life course, such as obesity^11^.

As regards environmental risk factors, the high pollen load in Łódź and PM concentration in Łódź and Thessaloniki could increase the risk for children respiratory health and allergic sensitization^13^. Porto and Rijeka could expose children to a higher level of gaseous pollutants. Regarding urbanization, children of Thessaloniki lived in a highly urbanized city. Pollen is often at the origin of rhinitis and asthma^2,13,14^. Air pollution is a significant public health problem and urbanization is firmly correlated to air pollution^15,16^. Recent Italian studies showed that grey spaces are related to adverse effects on children and adults allergic status and PAHs exposure in adulthood^17,18^. The beneficial influence of green space was shown for wellbeing, diastolic blood pressure, salivary cortisol, heart rate, incidence of diabetes, and total and cardiovascular mortality^19,20^. Meanwhile, a recent meta-analysis found that green space exposure is a risk factor for childhood wheeze, asthma, and allergic rhinitis^21^. Other studies have highlighted how urban green areas enhance human wellbeing by providing ecosystem services absent in grey areas. These include improving air quality, offering recreational opportunities, and enriching cultural experiences^22–24^. Additionally, green areas contribute to regulating local temperatures, reducing the urban heat island effect, and moderating adverse natural events like floods^22,25^. The latter are particularly important in light of potential changes in meteorological patterns, such as heat waves and extreme events, resulting from climate change^26^. European children and especially in the “climatic hot spot” of the Mediterranean area, will live in increasingly hot climates, with persistent heat waves. Substantial and abrupt variations in temperatures are related to detrimental health effects, such as increasing total mortality and allergic morbidity^2,25,26^. Furthermore, the rising temperature and carbon dioxide concentrations may impact on pollen allergenicity, seasonality, distribution, and load, thus increasing pollen exposure^13,14,26^. It is also supposed that higher temperatures are associated with faster rates of fungal growth (but with fewer spores) and that extreme events could increase indoor dampness and consequent fungal proliferation^2^. These changes, ultimately, might affect children’s allergic and respiratory health^2^.

Less known factors were also considered. Although likely underestimated, chronic exposure to noise pollution negatively impacts physical and mental health and wellbeing. It has been estimated that 113 million people are exposed to Lden of at least 55 dB from traffic and 22 million from railways in Europe^27^. Safe drinking water is a fundamental human right and it is essential to health, especially for the most vulnerable categories, such as infants, children, elderly and frail people^28^. The increasing frequency and severity of droughts may affect both the quality and quantity of water^28^. Lead exposure was associated with neurodevelopmental disorders, impaired renal function, hypertension, impaired fertility, adverse pregnancy outcomes, and mortality^28^. Animal studies showed a likely toxic effect on male reproductive tract of boron compounds exposure^28^. Even bacterial contamination may be dangerous for human health. Short-term peaks in pathogen concentration may considerably increase disease risks and expose the community to the risk of intestinal and other infectious disease outbreaks^28^.

Using plant protection products on crops or food products may result in pesticides residues in food, potentially posing a risk to public health^29^. To ensure a high level of consumer protection, Regulation (EC) No 396/20,059 established a legal limit of 0.01 mg/kg (MRLs)^29^. The higher percentage of MRLs exceeding in Portugal and Greece than in other countries generates concerns, which need to be deepened^29^.

### Comparison with EU and international limits

**Figure 2:**
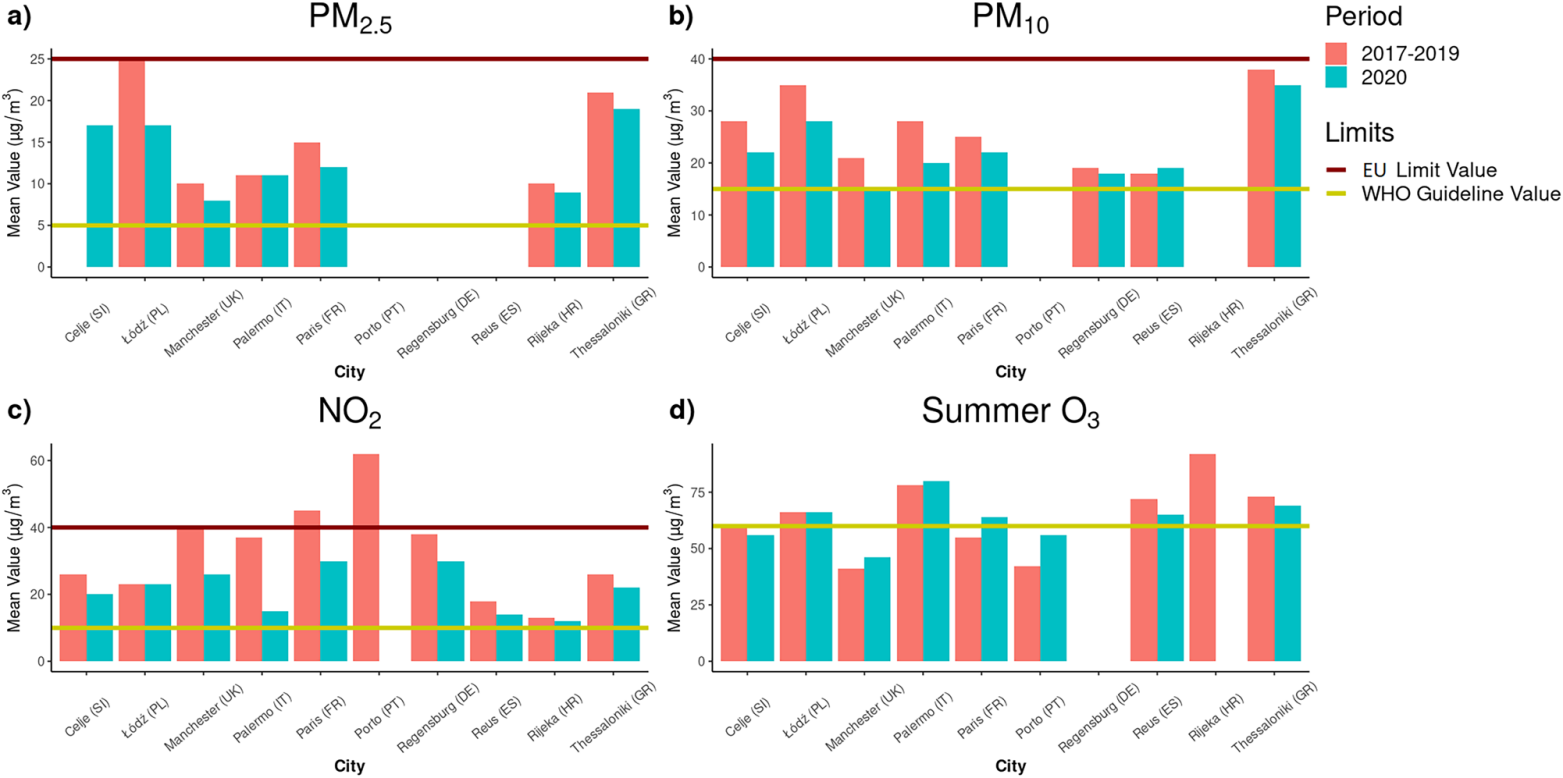
Mean values of air pollutants (PM_2.5_, PM_10_, NO_2_, summer O_3_) over the period 2017–2019 (orange) and 2020 (blue) for the ten cities, and the EU limits (red line) and WHO guidelines values (yellow line). PM_2.5_: particulate matter with an aerodynamic diameter smaller than 2.5 µm; PM_10_: particulate matter with an aerodynamic diameter smaller than 10 µm; NO_2_: Nitrogen Dioxide; summer O_3_: April-September Ozone; µg/m^3^: microgram/cubic meter; SI: Slovenia; PL: Poland; UK: United Kingdom; IT: Italy; FR: France; PT: Portugal; DE: Germany; ES: Spain; HR: Croatia; GR: Greece.

Currently, EU limits and WHO guidelines exist only for air pollution, noise and water parameters (Table 3). The differences between the EU air quality standards and the WHO recommended values have increased after the publication of the new WHO air quality guidelines (AQG) on September 22, 2021, indicating significantly lower levels than previous ones^30,31^. In the study period 2017–2019, the cities generally exceeded the PM and NO_2_ annual reference values of WHO air quality guidelines, but not of EU limits (Fig. 2, Table 3).

**Table 3:** References value from European Union Directive (EU) and international guidelines (WHO).

	EU		WHO	
	Directive	Limit value	Reference	Guideline’s value
**Air quality**				
PM_10_ (annual average, µg/m^3^)	2008/50/EC^30^	40	WHO, 2021^31^	15
PM_2.5_ (annual average, µg/m^3^)		25		5
NO_2_ (annual average, µg/m^3^)		40		10
O_3_ (summer average, µg/m^3^)		No limit value		60^a^
**Traffic noise**				
Lden (annual average, dB)	2002/49/EC^32^	55*	WHO, 2018^33^	53
Lnight (annual average, dB)		50*		45
**Railway noise**				
Lden (annual average, dB)	2002/49/EC^32^	55*	WHO, 2018^33^	54
Lnight (annual average, dB)		50*		44
**Drinking water**				
Acrylamide (µg/L)	2020/2184/EU^34^	0.1	WHO, 2022^28^	0.5
Antimony (µg/L)		10		20
Arsenic (µg/L)		10		10
Boron (mg/L)		1.5		2.4
Bromate (µg/L)		10		10
Cadmium (µg/L)		5		3
Chlorates and chlorites (mg/L)		0.25		0.7
Chromium (µg/L)		25		50
Copper (mg/L)		2		2
Cyanide (µg/L)		50		70^b^
Fluoride (mg/L)		1.5		1.5
Lead (µg/L)		5		10
Mercury (µg/L)		1		6
Nickel (µg/L)		20		70
Nitrate (mg/L)		50		50
Nitrite (mg/L)		0.50		3
Selenium (µg/L)		20		40
1,2-dichloroethane (µg/L)		3		30
Benzene (µg/L)		1		10
Benzo(a)pyrene (µg/L)		0.01		0.7
Epichlorohydrin (µg/L)		0.10		0.4
Haloacetic acids (HAA) (µg/L)		60		
Polycyclic aromatic hydrocarbons (PAHs) (µg/L)		0.1		
Tetrachloroethene and trichloroethene (µg/L)		10		8
Total Trihalomethanes (TTHMs) (µg/L)		100		
Vinyl chloride (µg/L)		0.5		0.3
*Enterococci* (CFU/100 ml)		0		0
*Escherichia coli* (CFU/100 ml)		0		0
Total pesticides (µg/L)		0.5		

^a^average of daily maximum 8-h mean O_3_ concentration in the six consecutive months with the highest running-average O_3_ concentration; ^b^referred to the precedent edition of WHO Guideline value; *no limit value but general thresholds indicating high noise levels; references in bibliography. Empty cells indicate that the guideline value was not provided.

Only Paris and Porto violate the NO_2_ EU limits. Despite lacking a summer limit for O_3_, the WHO air quality guidelines provided a seasonal recommended value, consisting of the average daily maximum 8-h mean O_3_ concentration in the six consecutive months with the highest average O_3_ concentration (60 µg/m^3^). Apart from Manchester, Paris and Porto, all the cities exceeded the O_3_ recommended value, in 2017–2019.

The EU Directive 2002/49/EC established general high noise thresholds: 55 dB for Lden and 50 dB for Lnight. The WHO European office defined lower guidelines values, based on the evidence on health effects (Table 3)^32,33^. Since exposed population percentage was provided according to EU limits, it is only possible to evidence their exceeding in almost every city.

Regarding drinking water, the substances above the EU limit and/or the WHO guidelines were: the 2017–2019 lead in Paris and Thessaloniki; the 2020 boron, and chlorates and chlorites in Celje and Łódź, respectively^28,34^. The presence of lead may be due to corrosive water effects on household plumbing systems containing lead in pipes or from the connections service to homes^28^. The amount of lead dissolved in the plumbing system depends on several physicochemical water properties^28^. Boron presence depends on the surrounding geology and wastewater discharges^28^. Values exceeding WHO guidelines and EU limits value of *Enterococci* and *Escherichia coli* were found in Porto drinking water. Celje drinking water had exceeded in *Escherichia coli* limits value, as well. The presence of bacteria in water is likely due to faecal contamination^28^.

### SARS-CoV-2 pandemic effects

As expected, the average mortality in 2020 among the ten cities increased, probably due to SARS-CoV-2 pandemic. Overall, the Covid-19 pandemic affected urban air pollution, especially PM and NO_2_ (Table 2). In Europe during Covid-19 lockdowns, road transport reductions caused a temporary drop in NO_2_ annual mean concentrations, up to 25% in France, Italy and Spain^35^. During the first lockdown in April 2020, the traffic monitoring stations registered a NO_2_ concentrations drop up to 70%^35^. However, the Covid-19 lockdown measures’ impact on the annual mean level of PM_10_ and PM_2.5_ was limited, and no greater than a median reduction of 4% and 5% across all stations, respectively^35^. This may be because increased emissions from residential heating could have counterbalanced reductions in other sectors^35^.

### Usefulness and feasibility

The EDMS has been implemented to provide an informative, interactive, user-friendly tool to support the scientific community and stakeholders in environmental-health studies and environmental policies. Recently, a growing interest in the role of the external exposome in disease development has led to an increasing number of studies on this topic^4,5^. The challenge remains finding and processing of these different data. In this paper, all the possible data that could be recovered, regarding external non-specific and specific exposome factors, has been archived. The added value of this work is that the EDMS can be further expanded and constantly updated to be used in other European settings dealing with lifetime external exposome.

However, its effectiveness is hampered by several criticisms. The difficulties in building and implementing the EDMS ranged from finding the data source to heterogeneity in the used language. In the Supplementary Information (Tables S2-S4) data search issues and source links are detailed.

One of the major concerns experienced for retrieving most environmental and lifestyle factors is the lack of a centralized database at the European level. This leads to time consuming data searches in separate national or even local sites or reports. Language barriers further exacerbate this issue, as many databases and reports are only accessible in the native language, thus requiring extensive translation efforts. Additionally, on web platforms exclusively in the native language, direct translation features within browsers were not always accessible, further complicating the translation process. Consequently, this linguistic harmonization has significantly consumed both time and staff resources, decreasing operational efficiency and productivity. Furthermore, the availability of reports rather than databases has necessitated manual extraction of data, so increasing the risk of human error and further prolonging the data retrieval process.

Another critical issue was the lack of standardization in reporting the data (e.g. different temporal resolution, parameters, unit of measures, etc.), which did not allow appropriate comparability of the various exposome factors among countries/cities.

Notwithstanding the existence of the climatic, air and noise pollution, and LU/LC European centralized databases (ECA&D, EEA and Copernicus databases), data for each of the ten studied cities were not always available. In many cases, data had insufficient coverage and therefore were unusable. In particular, this problem arose in air pollution data. The failure of European oversight of data supply, availability and quality or non-compliance of countries with European indications/regulations, results in a severe gap for multi-centre studies in this sector.

In addition, even if available, data did not completely cover the study period, weighting on the estimates of the various indicators. This aspect highlights Europe’s digital gap, which is expected to increase without faster and more comprehensive engagement in Artificial Intelligence (AI) technologies, especially in European countries with relatively low AI facilities.

Although the EU encourages the implementation of Open Data, and the adoption of the FAIR data principle, according to which data have to meet principles of findability, accessibility, interoperability, and reusability, public data availability remains limited^10^. Indeed, we have found the lower availability for open pollen data in most of the European countries in this paper. A public consultation of pollen data would be necessary in a world of increasing allergic population^13^.

Finally, regarding drinking water, not all parameters regulated by the European Union are provided in the local reports/databases, whereas it is the citizen's right to exhaustively know the most important characteristics of the supplied water.

Overall, Open Access datasets provide ecological data. In epidemiology, ecological studies are used to understand the relationship between health outcome and exposure at a population level, where the population represents a group of individuals with shared characteristics such as geography, ethnicity and socio-economic status. Ecologic studies are more often subject to confounding bias than analytic risk studies due to the lack of information on potential confounders. However, mixing specific and generic exposome can reduce such confounding bias.

In summary, the limitations in data accessibility, standardization, and spatial and temporal coverage pose significant challenges. Addressing these issues is crucial for enhancing the effectiveness of EDMS, enabling the integration of additional data on factors like pollution and socio-economic status. Our study was limited to a descriptive analysis, lacking statistical inferences and validation of the tool's effectiveness. Consequently, further research on the application of EDMS in environmental epidemiology is warranted. Nevertheless, we believe EDMS can be an excellent tool to support ecological studies by centralizing all exposures within a unified database. This not only streamlines researchers' efforts but also enhances the precision of exposome estimations. By overcoming data heterogeneity and enhancing spatial resolution of data, EDMS would facilitate comparisons of exposomes across various European populations, thereby aiding in the identification of population-specific risk factors. Consequently, policymakers are empowered to make prompt evidence-based decisions to mitigate the public health burden.

## Conclusions

We assessed external exposome by implementing an Environmental Data Management System, the EDMS, in ten European cities for the first time, ensuring the accessibility and the usability of data. As expected, a wide heterogeneity of risk factors exposures was shown across cities, highlighting some critical public health issues. From our study emerged the potential of standardized data management systems to facilitate interdisciplinary collaboration and data-driven decision-making in public health. Leveraging Open-Access databases for health research can yield numerous public health benefits including enhanced accessibility, accelerated discovery, transparency and reproducibility, cost-effectiveness, long-term sustainability, and finally ethical considerations (data sharing practices, research benefits equitably distributed across diverse populations). In conclusion, by harnessing these data management systems, we can address critical public health issues and pave the way for tailored preventive measures at the population level, thus ensuring a healthier future for generations to come.

### Supplementary Information

Supplementary Information.

## Data Availability

The datasets generated and analysed during the current study are available from the corresponding author on reasonable request.
