# Supplementary material for: Assessing external exposome by implementing an Environmental Data Management System using Open Data
Source: Sci Rep. 2024 Jul 26;14:17142. doi: 10.1038/s41598-024-62924-0 (PMC11282278; doi:10.1038/s41598-024-62924-0)
Supplement: Supplementary file 1 — Supplementary Information. [file 41598_2024_62924_MOESM1_ESM.pdf]

Assessing external exposome by implementing an Environmental Data Management System using Open Data

Sofia Tagliaferro, Sara Maio, Federico Pirona, Ilaria Stanisci, Giuseppe Sarno, Patrizia Silvi, Marianthi Kermenidou, Nafsika Papaioannou, Reena Perchard, Igor Prpic, Kinga Polanska, Joanna Jerzynska, Elisabete Ramos, Joaquim Rovira, Jordina Belmonte, Janja Snoj Tratnik, Milena Horvat, David Kocman, Zdravko Spiric, Jacqueline Zickella, Salvatore Fasola, Stefania La Grutta, Velia Malizia, Laura Montalbano, EarlyFOOD, HEALS EXHES & Sandra Baldacci, Isabella Annesi-Maesano

		Spatial resolution	Temporal resolution	Selection criteria	Statistics	Comment
EXTERNAL NON-SPECIFIC FACTORS						
**Socio-demographic indicators**						
**variable**	**definition**					
Population	Number of inhabitants on 1 January	City level	Annual mean data		The mean value of population (total number (n) and by sex (%)) was calculated over the period 2017-2020. The mean mortality rate ([mean deaths/mean population*1000] (%)) was calculated over 2017-2019, and the 2020 values were singly reported.	Rijeka population was reported by the National Statistical Institute on 31 December. So, to compare Rijeka's results with the other cities, data from 2016 to 2019 were taken. Regensburg mortality data were not found.
Mortality	Number of deaths					
Life expectancy at birth	The average life duration starting from the birth by sex	Country level	Annual mean data		The mean life expectancy at birth (mean age) by sex was calculated over the period 2017-2020. The mean population at risk of poverty was calculated over 2017-2019, and the 2020 values were singly reported.	
Population at risk of poverty	The share of persons whose equivalised income is below a certain threshold, computed according to national income distribution					
**Lifestyle risk factors**						
Body Mass Index (BMI, kg/m^2)	Population percentage underweight (< 18.5), normal weight (≥18.5 and < 25), pre-obese (≥25 and < 30), obese (≥ 30)	Country level	Annual mean data		The frequencies (%, percentage) of BMI, alcohol consumption, drug consumption and smoking were reported for the year of the specific surveys.	Information from National Statistics Institutes was not harmonized among countries (e.g. drug consumption was measured in terms of frequency or economics depending on the country). In 2020 the United Kingdom officially exited from the European Union ("Brexit"), causing the lacking participation in the 2019 European surveys.
Alcohol consumption	Population percentage consuming alcohol every day, every week, every month, less than once a month, or never					
Drug consumption	Population percentage of self-reported non-prescribed and prescribed drugs use in the last two weeks					
Smoking prevalence	Population percentage of smoking					
Climatic parameters						
Temperature	Monitoring network temperature data in Celsius degree (°C)	City level	Daily (datasets) or annual (annual reports) mean data	The monitoring stations within the city's municipalities boundaries were selected.	Firstly, for each station, the annual mean of the climatic parameters was calculated when not already available. When multiple stations were available for a city, the annual average of the climatic parameters measured by all stations within the city's municipality was calculated. Secondly, the average over the period 2017-2020 was calculated.	For Łódź, Manchester, Palermo and Thessaloniki, data from the local or national air quality monitoring networks were used, due to missing climatic data in the European central database (ECA&D).
Relative humidity	Monitoring network relative humidity data in percentage (%)					
Land Use/Land cover (LU/LC)						
44 land cover classes (Level 3) of the Corine Land Cover (CLC) inventory	Classes representing geographical information about the cover of artificial surfaces, agricultural land, forests and semi-natural areas, wetlands, and water bodies in Europe	City agglomeration level	Annual data	Urban Atlas (UA) LU/LC data for the year 2018 were used to extract agglomeration city's boundaries.[1]	The CLC and UA shapefiles were uploaded on Quantum-Geographical Information System (Q-GIS), free and open-source software to manage, edit and analyse environmental data.[2] First, a basic editing geoprocessing tool was used: the CLC layer was clipped with the city agglomeration boundaries layer from UA. Then, CLC classes' areas were recalculated on the agglomeration areas and the percentages were obtained. Finally, percentages were grouped into four principal classes: blue, green, grey, and agricultural areas according to Parmes et al.[3]	The UA shapefile was not available for the city of Celje. For this reason, it wasn't possible to calculate the percentage of CLC classes of that city.
EXTERNAL SPECIFIC FACTORS						
Air quality						
PM_{10}	Particulate matter with an aerodynamic diameter smaller than 10 µm in microgram/cubic meter	City level	Monthly (dataset) or annual (datasets/ reports) mean data	The monitoring stations within the cities' municipality boundaries and with a minimum	PM_{10}, $PM_{2.5}$ and NO_2 annual mean and O_3 summer mean (April-September) concentrations in microgram/cubic meter ($\mu g/m^3$) were calculated for each year and each monitoring station, when not	For Manchester, data from the local air quality monitoring network were used because some pollutants data

$PM_{2.5}$	Particulate matter with an aerodynamic diameter smaller than 2.5 µm in microgram/cubic meter			data coverage of 75% of valid data (as reported in the EEA Report 2020) were selected.[4]	already available from the annual datasets/reports. Afterwards, the averages over the period 2017-2019 of each air pollutant for each city were estimated. The 2020 values were singly reported.	were missing in the European central database (EEA).
NO_2	Nitrogen dioxide in microgram/cubic meter					
O_3	Ozone in microgram/cubic meter					
Pollen and spores						
Pollens and spores		City level	Daily (datasets) or weekly (reports) mean data	According to D'Amato et al. and Cecchi et al., pollen and spores of major allergy interest in Europe were selected: Asteraceae (*Ambrosia* and *Artemisia*), Betulaceae (*Alnus* and *Betula*), Corylaceae (*Carpinus* and *Corylus*), Cupressaceae, Fagaceae (*Fagus* and *Quercus*), Oleaceae (*Olea*), Poaceae, and Urticaceae in pollen/cubic meter; *Alternaria* and *Cladosporium* in spores/cubic meter.[5,6]	Firstly, the Annual Pollen Integral (APIn, annual sum of daily pollen concentrations, in pollen/cubic meter, p/m^3) was calculated for each pollen, city and year. When multiple stations were available for a city, the annual average of the APIn was calculated. Secondly, the pollen's APIn of each year were averaged over the period 2017-2020.<br>Moreover, in order to give a full overview at the local level, a cumulative Allergenic Pollen Integral (cAPI) was calculated. The mean APIn over 2017-2020 of the major allergenic pollen set (only the families of pollen reported in Table 2) were summed, as reported by the Italian Institute for Environmental Protection and Research (ISPRA) report.[7]<br>Regarding the spores, the annual sum of daily spores concentrations (spores/cubic meter, s/m^3) was calculated. Then, the annual sums were averaged over the period 2017-2020.	Except for Celje, Palermo, Rijeka and Thessaloniki, data were not public. So, it was usually necessary to contact the HEALS and EarlyFOOD partners directly to request the pollen/spores information from the local biomonitoring services. For Palermo, data from 2017 to 2019 were lacking due to the delayed installation of the monitoring station. For Celje, Reus and Manchester, data were available for the nearest cities of Ljubljana, Tarragona and Chester, respectively.
Noise						
Population exposure by traffic, railway, airports, industries sources	Number of people in urban agglomerations exposed to annual day-evening-night-weighted sound pressure levels (Lden) and annual night-weighted sound pressure levels (Lnight)	City agglomeration level	Annual mean data	Only data on noise exposure from major roads and railways were selected, because data from major airports and industries was scarce.	Noise bands provided by EEA (Lden: 55-59, 60-64, 65-69, 70-74, >75; Lnight: 50-54, 55-59, 60-64, 65-69, >70) were grouped, reporting the percentage of the population exposed and not exposed to cut-off: 55 dB for Lden, 50 dB for Lnight.	Celje is not included in the European city agglomerations which provided noise data.
Drinking water						
Physicochemical and biological parameters of water quality of aqueducts	Physicochemical and biological parameters concentration measured through ad hoc monitoring campaign	City level	Annual mean data (datasets, reports) or quarterly mean data (reports)	The main physicochemical and biological parameters were selected as suggested by the EU Directive 2020/2184 on the quality of water.[8] The maximum value for each year/parameter was considered because it was the only metric available to compare the parameters among the cities. When the values were reported as less than the limit of quantification in the datasets/reports, we set them as the half of the value of limit of quantification, as reported by the Directive (EC) 2009/90.[9]	The average values of the maximum concentration of drinking water quality parameters over the periods 2017-2019 and 2020 were calculated. The values that resulted below the limits of quantification were reported as below the relative limit of quantification of each city's water agency (in Table 2, as "<" than the relative limit).	The values do not reflect reality, but only an estimate of the average exposure of the population to these parameters, for descriptive purposes only.
Food pesticides						
Maximum Residue Levels (MRLs) of pesticides in food	Legal limits established in the European Regulation (EC) No 396/2005 in order to ensure the minimum possible exposure of the EU consumers and to protect vulnerable subjects. The European Food Safety Authority - EFSA provided the percentage of the pesticides MRLs in food for EU Member States, Iceland and Norway. These limits are set for more than 1300 pesticides covering 378 food products/food groups.[10]	Country level	Annual data		The annual percentage of MRLs for the ten countries was collected and then the mean over the study periods 2017-2019 was calculated. The 2020 values were singly reported.	

Table S1. Characteristics of data and methods applied to construct exposome variables.

	Celje<br>(Slovenia)	Łódź (Poland)	Manchester<br>(United Kingdom)	Palermo<br>(Italy)	Paris<br>(France)	Porto<br>(Portugal)	Regensburg<br>(Germany)	Reus<br>(Spain)	Rijeka<br>(Croatia)	Thessaloniki<br>(Greece)
EXTERNAL NON-SPECIFIC FACTORS										
**Socio-demographic indicators**										
Source	SIStat<br><br>EU-SILC survey (population at risk of poverty)	GUS<br><br>EU-SILC survey (population at risk of poverty)	ONS<br><br>EU-SILC survey (population at risk of poverty)	Istat<br><br>EU-SILC survey (population at risk of poverty)	Insee<br><br>EU-SILC survey (population at risk of poverty)	PORDATA<br><br>EU-SILC survey (population at risk of poverty)	Destatis<br><br>EU-SILC survey (population at risk of poverty)	INE<br><br>EU-SILC survey (population at risk of poverty)	DZS<br>(population and mortality)<br><br>World Bank<br>(life expectancy)<br><br>EU-SILC survey (population at risk of poverty)	ELSTAT<br>(population, mortality, and life expectancy 2017)<br><br>World Bank<br>(life expectancy 2018-2020)<br><br>EU-SILC survey (population at risk of poverty)
Accessibility	Open data	Open data	Open data	Open data	Open data	Open data	Open data	Open data	Open data	Open data
Modality	Database	Database	Database	Database	Database	Database	Database	Database	Database	Database
Site language	Slovenian/English	Polish/English	English/Cymraeg (Welsh)	Italian/English	French/English	Portuguese/English	German/English	Catalan/English	Croatian/English	Hellenic/English
Data language	Slovenian/English	Polish/English	English	Italian/English	French/English	Portuguese/English	German	Catalan/English	Croatian/English	Hellenic/English
**Life-style risk factors**										
Source	Eurostat	Eurostat	Eurostat<br><br>NHS Digital (BMI)	Eurostat	Eurostat	Eurostat	Eurostat	Eurostat	Eurostat	Eurostat
Accessibility	Open data	Open data	Open data	Open data	Open data	Open data	Open data	Open data	Open data	Open data
Modality	Database	Database	Database	Database	Database	Database	Database	Database	Database	Database
Site language	English/German/ French	English/German/ French	English/German/ French	English/German/ French	English/German/ French	English/German/ French	English/German/ French	English/German/ French	English/German/ French	English/German/ French
Data language	English/German/ French	English/German/ French	English/German/ French	English/German/ French	English/German/ French	English/German/ French	English/German/ French	English/German/ French	English/German/ French	English/German/ French
Climatic parameters										
Source	ECA&D	IMGW	CEDA	SCIA - ISPRA	ECA&D	ECA&D	ECA&D	ECA&D	ECA&D	HNMS
Accessibility	Open data	Open data	Open data with registration	Open data	Open data	Open data	Open data	Open data	Open data	Open data
Modality	Database	Online map	Database	Database	Database	Database	Database	Database	Database	Monthly report
Site Language	English	Polish	English	Italian	English	English	English	English	English	Hellenic/English
Data language	English	Polish	English	Italian	English	English	English	English	English	English
Land use/ land cover										
Source		Copernicus Programme	Copernicus Programme	Copernicus Programme	Copernicus Programme	Copernicus Programme	Copernicus Programme	Copernicus Programme	Copernicus Programme	Copernicus Programme
Accessibility		Open data with registration	Open data with registration	Open data with registration	Open data with registration	Open data with registration	Open data with registration	Open data with registration	Open data with registration	Open data with registration
Modality		Spatial data	Spatial data	Spatial data	Spatial data	Spatial data	Spatial data	Spatial data	Spatial data	Spatial data
Site language		English/French/ German	English/French/ German	English/French/ German	English/French/ German	English/French/ German	English/French/ German	English/French/ German	English/French/ German	English/French/ German
Data language		English	English	English	English	English	English	English	English	English
EXTERNAL SPECIFIC FACTORS										
Air quality										
Source	EEA	EEA	Clean Air GM	EEA	EEA	EEA	EEA	EEA	EEA	EEA
Accessibility	Open data	Open data	Open data	Open data	Open data	Open data	Open data	Open data	Open data	Open data
Modality	Database	Database	Annual report	Database	Database	Database	Database	Database	Database	Database
Site Language	English	English	English	English	English	English	English	English	English	English
Data language	English	English	English	English	English	English	English	English	English	English

Pollen and spores										
Source	ARSO	Medical University of Lodz	Met Office	ARPA Sicilia	RNSA			Xarxa Aerobiològica de Catalunya	Nastavni Zadov Za Javno Zdravstvo	Envdimosthes
Accessibility	Open data	On request	On request	Open data	On request			On request	Open data	Open data
Modality	Monthly report	Database	Database	Weekly report	Database			Database	Annual report	Database
Site language	Slovenian/English		English	Italian	French/English			Catalan/Spanish/ English	Croatian	Hellenic
Data language	Slovenian/English	English	English	Italian	French			English	Croatian	English
Noise										
Source		EEA	EEA	EEA	EEA	EEA	EEA	EEA	EEA	EEA
Accessibility		Open data	Open data	Open data	Open data	Open data	Open data	Open data	Open data	Open data
Modality		Database	Database	Database	Database	Database	Database	Database	Database	Database
Site language		EU languages	EU languages	EU languages	EU languages	EU languages	EU languages	EU languages	EU languages	EU languages
Data language		English	English	English	English	English	English	English	English	English
Drinking water										
Source	VOKA d.o.o.	ZWIK sp. z o.o.	Manchester Water Works	AMAP S.p.A.	data.gouv.fr	Águas e Energia do Porto		Aigües de Reus	Nastavni Zadov Za Javno Zdravstvo	EYATH
Accessibility	On request	Open data	Open data	Open data	Open data	Open data		Open data	Open data	Open data
Modality	Annual report	Online table	Annual report	Online table	Database	Quarterly reports		Quarterly reports	Annual report	Online table
Site language	Slovenian	Polish	English	Italian/English	French/English/Spanish	Portuguese		Catalan/Spanish	Croatian	Hellenic/English
Data language	Slovenian	Polish	English	Italian/English	French	Portuguese		Catalan	Croatian	Hellenic/English
Food pesticides										
Source	EFSA	EFSA	EFSA	EFSA	EFSA	EFSA	EFSA	EFSA	EFSA	EFSA
Accessibility	Open data	Open data	Open data	Open data	Open data	Open data	Open data	Open data	Open data	Open data
Modality	Annual report	Annual report	Annual report	Annual report	Annual report	Annual report	Annual report	Annual report	Annual report	Annual report
Site language	English	English	English	English	English	English	English	English	English	English
Data language	English	English	English	English	English	English	English	English	English	English

Table S2. Characteristics of data retrieval for external non-specific and specific exposome for the ten cities contributing to HEALS and EarlyFOOD projects.

SIstat: Statistični urad Republika Sloveija; GUS: Główny Urząd Statystyczny; ONS: Office for National Statistics; Istat: Istituto nazionale di Statistica; Insee: Institut national de la statistique et des études économiques; PORDATA: Estatísticas sobre Portugal e Europa; Destatis: Statistisches Bundesam; INE: Instituto Nacional de Estadística; DZS: Državni zavod za statistiku; EU-SILC: EU statistics on income and living conditions; ELSTAT: Hellenic Statistical Authority; Eurostat: european statistics; NHS Digital: National Health Service; ECA&D: European Climate Assesment & Dataset; IMGW: Instytut Meteorologii i Gospodarki Wodnej; CEDA: Centre for Environmental Data Analysis; SCIA-ISPRA: Sistema nazionale per l'elaborazione e diffusione di dati climatici, Istituto Superiore per la Protezione e la Ricerca Ambientale; HNMS: Hellenic National Meteorological Service; EEA: European Environment Agency; Clean Air GM: Clean Air Greater Manchester; ARSO: Agencija Republike Slovenije za okolje; ARPA Sicilia: Agenzia Regionale per la Protezione dell'Ambiente Sicilia; RNSA: Réseau National de Surveillance Aerobiologique; Envdimosthes: Environmental Department, Municipality of Thessaloniki; VOKA d.o.o.: Vodovod Kanalizacija Celje, d.o.o.; ZWIK sp. z o.o.: Zakładu Wodociągów i Kanalizacji sp. z o.o.; AMAP S.p.A.: Azienda Municipalizzata Acquedotto di Palermo; EYATH: Thessaloniki Water Supply & Sewerage Company SA; EFSA: European Food Safety Authority.

	Celje (Slovenia)	Lodz (Poland)	Manchester (UK)	Palermo (Italy)	Paris (France)	Porto (Portugal)	Regensburg (Germany)	Reus (Spain)	Rijeka (Croatia)	Thessaloniki (Greece)
EXTERNAL NON-SPECIFIC FACTORS										
**Socio-demographic indicators**										
Population:										
Total	2017-2020	2017-2020	2017-2020[a]	2017-2020	2017-2020	2017-2020	2017-2020	2017-2020	2016-2019[b]	2017-2020[c]
By sex	2017-2020	2017-2020	2017-2020[a]	2017-2020	2017-2020	2017-2020	2017-2020	2017-2020	2016-2019[b]	2017-2020[c]
Mortality	2017-2020[d]	2017-2020	2017-2020	2017-2020	2017-2020	2017-2020		2017-2020	2017-2020	2017-2020[c]
Life expectancy at birth by sex*	2017-2020	2017-2020	2017-2020[e]	2017-2020	2017-2020	2017-2019	2017-2020[e]	2017-2020	2017-2020	2017-2020
Population at risk of poverty*	2017-2020	2017-2020	2017-2018	2017-2020	2017-2020	2017-2020	2017-2020	2017-2020	2017-2020	2017-2020
**Lifestyle risk factors***										
Smoking prevalence	2017,2020	2017,2020	2017,2020	2017,2020	2017,2020	2017,2020	2017,2020	2017,2020	2017,2020	2017,2020
BMI	2019	2019	2019	2019	2019	2019	2019	2019	2019	2019
Alcohol consumption	2019	2019		2019	2019	2019	2019	2019	2019	2019
Drugs consumption	2019	2019		2019	2019	2019	2019	2019	2019	2019
Climatic factors										
Temperature	2017-2020	2017-2020	2017-2020	2017-2020	2017-2020	2017-2020[f]	2017-2020	2017-2020	2017-2020	2017-2019, 2020[g]
Relative humidity	2017-2020	2017-2020[f]	2017-2020	2017-2020	2017-2020		2017-2020	2017-2020	2017-2020	
Land Use/Land cover										
Blue		2018	2018	2018	2018	2018	2018	2018	2018	2018
Grey		2018	2018	2018	2018	2018	2018	2018	2018	2018
Green		2018	2018	2018	2018	2018	2018	2018	2018	2018
Agricultural		2018	2018	2018	2018	2018	2018	2018	2018	2018
EXTERNAL SPECIFIC FACTORS										
Air quality										
PM_{10}	2017-2020	2017-2020	2017-2020	2017-2018, 2019[f], 2020	2017-2020	2017-2019[f]	2017-2020	2017-2020	2017-2018[f]	2017[f], 2018-2020
$PM_{2.5}$	2020	2017-2020	2017-2020	2018, 2019[f], 2020	2017-2020	2018-2019[f]			2017-2020	2017-2018[f],2019-2020
NO_2	2017-2020	2017-2020	2017-2020	2017-2020	2017-2020	2017[f],2018, 2019[f]	2017-2020	2017-2020	2017-2020	2017-2018[f], 2019-2020
O_3	2017-2020	2017-2020	2017-2020	2017-2020	2017-2020	2017, 2018-2019[f], 2020		2017-2020	2017-2019, 2020[f]	2017,2018[f], 2019-2020
Pollen and spores										
Pollen										
Betulaceae:										
Alnus	2017-2020[h]	2017-2020	2017-2020[h]	2020	2017-2020			2017-2020[h]	2018-2020	2017,2019,2020
Betula	2017-2020[h]	2017-2020	2017-2020[h]	2020	2017-2020			2017-2020[h]	2017-2020	2017,2019,2020
Compositae:										
Ambrosia	2017-2020[h]	2017-2020	2017-2020[h]	2020	2017-2020				2017-2020	2017-2020
Artemisia	2017-2020[h]	2017-2020	2017-2018[h]	2020	2017-2020			2017-2020[h]		2017-2020
Corylaceae:										
Carpinus		2017-2020		2020	2017-2020				2017-2020	2017,2019,2020
Corylus	2017-2020[h]	2017-2020	2017-2020[h]	2020	2017-2020			2017-2020[h]	2018-2020	2017,2019,2020
Cupressaceae	2017-2020[h]			2020	2017-2020			2017-2020[h]	2017-2020	2017-2020
Fagaceae:										
Fagus	2017-2020[h]	2017-2020		2020	2017-2020			2017-2020[h]	2020	2017
Quercus	2017-2020[h]	2017-2020	2017-2020[h]	2020	2017-2020			2017-2020[h]	2017-2020	2017,2019,2020
Poaceae	2017-2020[h]	2017-2020	2017-2020[h]	2020	2017-2020			2017-2020[h]	2017-2020	2017-2020
Oleaceae:				2020				2017-2020[h]		2018-2020
Olea	2017, 2018[h]			2020	2017-2020			2017-2020[h]	2017-2020	2017
Urticaceae	2017-2020[h]	2017-2020	2017-2020[h]	2020	2017-2020			2017-2020[h]	2017-2020	2017-2020
Spores										

Alternaria		2017-2020		2020	2017-2020			2017-2020[h]		2017-2020
Cladosporium		2017-2020			2017-2020			2017-2020[h]		2017-2020
Noise										
Traffic:										
Lden		2017	2017	2017	2017	2017	2017	2017	2017	
Lnight		2017	2017	2017	2017	2017	2017	2017	2017	
Railway:										
Lden		2017	2017	2017	2017	2017	2017	2017	2017	
Lnight		2017	2017		2017	2017	2017	2017	2017	
Drinking water										
1,2-dichloroethane	2017-2020	2020			2017-2020	2017-2020			2019	2019
Acrylamide						2017-2020			2019	
Antimony	2017-2020	2020			2017-2020	2017-2020			2019	2019
Arsenic	2017-2020	2020			2017-2020	2017-2020			2019	2019
Benzene	2017-2020	2020			2017-2020	2017-2020			2019	2019
Benzo(a)pyrene	2017-2020	2020			2017-2020	2017-2020			2019	2019
Boron	2017-2020	2020			2017-2020	2017-2020			2019	2019
Bromate	2020		2017,2019		2017-2020	2017-2020			2019	2019
Cadmium	2017-2020	2020		2018	2017-2020	2017-2020		2017-2020	2019	2019
Chlorates and chlorites		2020								
Chromium	2017-2020	2020		2018	2017-2020	2017-2020		2017-2020	2019	2019
Copper	2017-2020	2020	2017-2020		2017,2018	2017-2020		2017-2020	2019	2019
Cyanide	2020	2020			2017-2020	2017-2020			2019	2019
Epichlorohydrin						2017-2020			2019	
Enterococci	2017-2020	2020		2018	2017-2020	2018-2020		2017,2019,2020	2019	
Escherichia coli	2017-2020	2020		2018	2017-2020	2017-2020		2017-2020	2019	2019
Fluoride	2017-2020	2020	2017-2020	2018	2017-2020	2017-2020		2017-2020	2019	2019
Haloacetic acids			2017-2020							
Lead	2017-2020	2020	2017-2020	2018	2017-2019	2017-2020		2017-2020	2019	2019
Mercury	2017-2020	2020			2017-2020	2017-2020			2019	2019
Nickel	2017-2020	2020		2018	2017	2017-2020		2017-2020	2019	2019
Nitrate	2017-2020	2020		2018	2017-2020	2017-2020		2017-2020	2019	2019
Nitrite	2017-2020	2020		2018	2017-2020	2017-2020		2017-2020	2019	2019
Polycyclic aromatic hydrocarbons (PAH)	2017-2020	2020			2017-2020	2017-2020			2019	2019
Selenium	2017-2020	2020			2017-2020	2017-2020			2019	2019
Tetrachloroethene and Trichloroethene	2017-2020	2020			2017-2019	2017-2020			2019	2019
Total pesticides	2017-2020	2020			2017-2020	2017-2020				2019
Total Trihalomethanes (TTHMs)	2017-2020	2020	2017-2020		2017-2020	2017-2020				2019
Vinyl Chloride					2017-2020	2017-2020				
Food pesticides*										
Pesticides residues > MRL	2018-2020	2017-2020	2017-2020	2017-2020	2017-2020	2017-2020	2017-2020	2017-2020	2017-2020	2017-2020

Table S3. Data availability for the period 2017-2020 for each external non-specific and specific exposome factors for the ten cities contributing to HEALS and EarlyFOOD projects.

*Data at country level; [a]mid-year population; [b]data from 2016 to 2019 were taken, because population was reported on 31 December; [c]Thessaloniki data were referred to the Thessaloniki prefecture instead the city; [d]Celje mortality data were referred to Celje province instead the city; [e]life expectancy was provided as computed over two years before and the current year (e.g. for 2017, the life expectancy was computed over 2015-2017); [f]data coverage less than 75%; [g]data was omitted because data coverage was equal to 75%, but the missing data were concentrated in winter, heavily affecting the annual average; [h]data were referred to the nearest city with a monitoring station.

Sources	Country	Sources link	Copyright policies
EXTERNAL NON-SPECIFIC FACTORS			
Socio-demographic indicators			
SIstat: Statistični urad Republike Slovenije (SURS)<br><br>"Own calculation"	SI	https://www.stat.si/StatWeb/	Statistical data and other information published on www.stat.si are available royalty-free and are given for free use to all users, which means that they can be freely:<br>- used, copied, downloaded for personal use or for non-commercial and commercial purposes;<br>- published or in any other way communicated to the public;<br>on condition that whenever the data or information are used the Statistical Office of the Republic of Slovenia (or SURS) is acknowledged as their source.
GUS: Główny Urząd Statystyczny	PL	https://stat.gov.pl/	The Central Statistical Office (CSO):<br>- has no objection to copying files and pages and making printouts, including its own studies, provided the source is indicated,<br>- also has no objection to links being made via a hyperlink to a web page, provided the source of the material is indicated,<br>- is not responsible for the content of web pages linked to the CSO web pages or for the presentation of own compilations (changes of content) based on the CSO data.
ONS: Office for National Statistics<br><br>"This information is licensed under the Open Government Licence v3.0. https://www.nationalarchives.gov.uk/doc/open-government-licence/version/3/"	UK	https://www.ons.gov.uk/	Under the terms of the Open Government Licence (OGL) and UK Government Licensing Framework (launched 30 September 2010), anyone wishing to use or re-use ONS material, whether commercially or privately, may do so freely without a specific application for a licence, subject to the conditions of the OGL and the Framework.
Istat: Istituto nazionale di Statistica<br><br>"This information is licensed under the Creative Commons License – Attribution – 3.0."	IT	http://dati.istat.it/	Data and analysis from the Italian National Institute of Statistics can be copied, distributed, transmitted and freely adapted, even for commercial purposes, provided that the source is acknowledged. No permission is necessary to hyperlink to pages on this website.
Insee: Institut national de la statistique et des études économiques	FR	https://www.insee.fr/fr/accueil	The INSEE welcomes the broadest use of the database products of which it is editor and holder of intellectual property rights (copyright and producer's rights). To this effect, it authorizes the reuse of data (redistribution) for commercial use. This redistribution is subject or not, as the case may be, to the signing of a license and to payment of a fee.<br>The database products are gradually being made available to the public free of charge on the INSEE website, as well as on websites with which the INSEE is linked. These products are made available when the technical conditions and the regulations regarding information confidentiality so allow.
PORDATA: Estatísticas sobre Portugal e Europa	PT	https://www.pordata.pt/	PORDATA embodies one of the priorities of the Foundation: the collection, compilation, systematization and dissemination of data on multiple areas of society, for Portugal and its municipalities, and for the European countries. The reported statistics derive from official and certified sources, with data production skills in the respective areas. The Foundation's endeavor consists in collecting and organizing the data available, making it as clear and accessible as possible. The Foundation believes to be providing a public service to the Portuguese society, free of charge and without any cost to the user.
Statistisches Bundesamt (Destatis), "Genesis-Online; Data licence by-2-0 (www.govdata.de/dl-de/by-2-0); own calculation"	DE	https://www.destatis.de/DE/Home/_inhalt.html	The data and meta-data provided may, for commercial and non-commercial use, in particular:<br>1. be copied, printed, presented, altered, processed and transmitted to third parties;<br>2. be merged with own data and with the data of others and be combined to form new and independent datasets;<br>3. be integrated in internal and external business processes, products and applications in public and non-public electronic networks.
INE: Instituto Nacional de Estadística<br><br>"Own compilation with data taken from the INE website: www.ine.es"	ES	https://www.ine.es/index.htm	The information contained on this website comes from numerous sources and therefore, the INE only authorises the re-use of that information whose original source is the INE itself. This re-used may be for commercial or non-commercial purposes and will always be carried out under the following general conditions:<br>- denaturalising the sense of the information is expressly prohibited;<br>- the source of the re-used target information must be quoted. This quotation may be carried out as follows: Source: INE website: www.ine.es if the data is not processed or: Own compilation with data taken from the INE website: www.ine.es if the data is processed;<br>- this must mention the date of the latest updating of the target information re-used, so long as it is included in the original.<br>- it may not be indicated, implied or suggested that the INE participates, sponsors or supports the re-use carried out with the information;<br>The INE shall not be held responsible for the use made of the information by the re-use agents. The INE shall not be held responsible for material damages or damage to data, nor for possible economic damage caused by the use of the re-used information.
ELSTAT: Hellenic Statistical Authority<br><br>"Own calculation"	GR	https://www.statistics.gr/el/home	The Hellenic Statistical Authority (ELSTAT) encourages the free reuse of its data, both for non-commercial and commercial purposes. The content of web pages, the statistical data, the metadata and the statistical publications and other documents published on ELSTAT's website, with the exceptions listed below, can be reused without any payment or written licence, provided that:<br>- their source is indicated;<br>- when reuse involves any modification to the data or text, including the translation of text, this is stated clearly to the end user of the information, as well as that ELSTAT bears no responsibility for the result of the modification.
DZS: Državni zavod za statistiku	HR	https://podaci.dzs.hr/hr/	When using data, users are kindly requested to indicate the Croatian Bureau of Statistics as the source (https://dzs.gov.hr/info-corner/1077).<br>The right of access to information and re-use of information is exercised in accordance with the Act on the Right of Access to Information (Official Gazette, Nos 25/13, 85/15, 69/22) and Directive (EU) 2019/1024 of the European Parliament and of the Council of 20 June 2019 on open data and the re-use of public sector information.

			The reuse of information, pursuant to Article 5, paragraph 1, item 6 of the Act "means the use of information from a public authority by a physical or legal person, for commercial or non-commercial purposes, different from the original purpose, within the framework of the public work for which that information was created, which is performed within the legally prescribed scope or work that is usually considered public work. The exchange of information between public authorities for the purpose of performing the work within their competence shall not be deemed to be reuse".<br>Data users have the right to:<br>- copy, publish, distribute and communicate information to the public;<br>- process information;<br>- use information for commercial and non-commercial purposes, combining them with other information and including them into their product.<br>User obligations: indicate the data source. By taking over or downloading information for further use, users accept the specified terms of use.
The World Bank<br><br>"Data licence by Creative Commons Attribution 4.0 International License (CC BY 4.0), (https://www.worldbank.org/en/about/legal/terms-of-use-for-datasets); own calculation"		https://www.worldbank.org/en/home	The World Bank strives to enhance public access to and use of data that it collects and publishes. The data are organized in datasets listed in The World Bank Data Catalog (the "Datasets"). The World Bank provides you with access to the Datasets free of charge subject to the terms of this agreement (these "Dataset Terms"), and subject to the general Terms and Conditions for using the World Bank website, which are incorporated into these Dataset Terms.<br>You are encouraged to use the Datasets to benefit yourself and others in creative ways. You may extract, download, and make copies of the data contained in the Datasets, and you may share that data with third parties according to these terms of use.
Lifestyle risk factors			
Eurostat: European statistics.<br>EHIS Survey (European Health Interview Survey) and DG Sante study (Directorate-General for Health and Food Safety)<br><br>"Data licence by Creative Commons Attribution 4.0 International License (CC BY 4.0); own calculation"	EU	https://ec.europa.eu/eurostat/en/	The Commission's reuse policy is implemented by the Commission Decision of 12 December 2011 on the reuse of Commission documents. Unless otherwise indicated (e.g. in individual copyright notices), content owned by the EU on this website is licensed under the Creative Commons Attribution 4.0 International (CC BY 4.0) licence. This means that reuse is allowed, provided appropriate credit is given and changes are indicated.
NHS: Digital, National Health Service<br><br>"This information is licensed under the Open Government Licence v3.0. https://www.nationalarchives.gov.uk/doc/open-government-licence/version/3/"	UK	https://digital.nhs.uk/	Copyright and database rights in the NHS England Content are released free-of-charge under the current version of the Open Government Licence (OGL), except where specified, either in these terms and conditions, elsewhere on this website or in the OGL terms. This licence does not extend to any other intellectual property rights, including but not limited to patents, design rights and trade marks. If there is any conflict between the OGL terms and these terms and conditions shall take precedence.<br>This means that you can use NHS England Content, including copying it, adapting it, and using it for any purpose, including commercially, provided you follow these terms and conditions and the terms of the OGL.
Climatic parameters			
IMGW: Instytut Meteorologii i Gospodarki Wodnej	PL	https://meteo.imgw.pl/	The legal basis for data sharing is the Act of 11 August 2021 on Open Data and Re-use of Public Sector Information (Journal of Laws of 11 August 2021).<br>Everyone shall have the right to re-use public sector information.
CEDA: Centre for Environmental Data Analysis<br><br>"Met Office (2020): UK Daily Temperature Data, Part of the Met Office Integrated Data Archive System (MIDAS). NCAS British Atmospheric Data Centre, 2023 citation. http://catalogue.ceda.ac.uk/uuid/1bb479d3b1e38c339adb9c82c15579d8"	UK	https://catalogue.ceda.ac.uk/	Data from CEDA should be cited in any article or presentation making use of the data. A full citation, akin to academic papers, will be found on the dataset catalogue page.
SCIA-ISPRA: Sistema nazionale per l'elaborazione e diffusione di dati climatici - Istituto Superiore per la Protezione e la Ricerca Ambientale	IT	http://www.scia.isprambiente.it/wwwrootscia/Home_new.html#	Climatological data and products available through SCIA cannot be commercialized, reproduced, given to other parties, included or distributed through other WEB sites.<br>Communications and/or publications making use of SCIA data and products must quote the source.
HNMS: Hellenic National Meteorological Service	GR	http://www.emy.gr/emy/en/	Permission to data re-use was requested and confirmed by the competent authority.
ECA&D: European Climate Assessment & Dataset<br><br>"We acknowledge the data providers in the ECA&D project. Klein Tank, A.M.G. and Coauthors, 2002. Daily dataset of 20th-century surface air temperature and precipitation series for the European Climate Assessment. Int. J. of Climatol., 22, 1441-1453. Data and metadata available at https://www.ecad.eu"	EU	https://www.ecad.eu/	Whenever you publish research or applications based in whole or in part on these data, you should include the following citation and acknowledgement:<br><br>"We acknowledge the data providers in the ECA&D project. Klein Tank, A.M.G. and Coauthors, 2002. Daily dataset of 20th-century surface air temperature and precipitation series for the European Climate Assessment. Int. J. of Climatol., 22, 1441-1453. Data and metadata available at https://www.ecad.eu"
Land use/Land cover			

Copernicus Programme<br><br>"The data were produced with funding by the European Union".	EU	https://land.copernicus.eu/	Access to data is based on a principle of full, open and free access as established by the Copernicus data and information policy Regulation (EU) No 1159/2013 of 12 July 2013. This regulation establishes registration and licensing conditions for GMES/Copernicus users and can be found here: http://eur-lex.europa.eu/legal-content/EN/TXT/?uri=CELEX%3A32013R1159.<br>Free, full and open access to this data set is made on the conditions that:<br>1. when distributing or communicating Copernicus dedicated data and Copernicus service information to the public, users shall inform the public of the source of that data and information;<br>2. users shall make sure not to convey the impression to the public that the user's activities are officially endorsed by the Union;<br>3. where that data or information has been adapted or modified, the user shall clearly state this;<br>4. the data remain the sole property of the European Union. Any information and data produced in the framework of the action shall be the sole property of the European Union.
EXTERNAL SPECIFIC FACTORS			
Air quality			
Clean Air GM: Clean Air Greater Manchester	UK	https://cleanairgm.com/	Use of this data is subject to terms and conditions: You are granted a non-exclusive, royalty free, revocable licence solely to view the Licensed Data for non-commercial purposes for the period during which Transport for Greater Manchester makes it available; you are not permitted to copy, sub-license, distribute, sell or otherwise make available the Licensed Data to third parties in any form; and third party rights to enforce the terms of this licence shall be reserved to Ordnance Survey.
EEA: European Environment Agency.	EU	https://aqportal.discomap.eea.europa.eu/	Unless otherwise indicated, the European Environment Agency (EEA) is the owner of copyrights and database rights in this website and its contents. The sources and owner(s) of the content are clearly indicated for each content.<br>Information, documents and material available on this website and for which the EEA holds the rights of use are public and may be re-used without prior permission, free of charge, for commercial or non-commercial purposes, provided that the EEA is always acknowledged as the original source of the material and that the original meaning or message of the content is not distorted.
Pollen and spores			
ARSO: Agencija Republike Slovenije za okolje	SI	https://www.arso.gov.si/	Permission to data re-use was requested and confirmed by the competent authority.
ARPA Sicilia: Agenzia Regionale per la Protezione dell'Ambiente Sicilia	IT	https://www.arpa.sicilia.it/	Civic access (simple or generalized) allows anyone to access data, documents and information of public administrations without the need to demonstrate a legitimate interest (Article 5, Legislative Decrete 33/2013).
Envdimosthes, Environmental Department, Municipality of Thessaloniki (Τμήμα Περιβάλλοντος και Προσαρμογής στην Κλιματική Αλλαγή του Δήμου Θεσσαλονίκης).	GR	https://envdimosthes.gr/	Permission to data re-use was requested and confirmed by the competent authority.
Xarxa Aerobiològica de Catalunya	ES	https://aerobiologia.cat/pia/en/	The pollen data was provided on request by Dr. Jordina Belmonte. The site of the Xarxa Aerobiològica de Catalunya in Tarragona (Spain) is possible thanks to the financial support from Diputació de Tarragona. This research contributes to the "María de Maeztu" Programme for Units of Excellence of the Spanish Ministry of Science and Innovation (CEX2019-000940-M).
RNSA: Réseau National de Surveillance Aerobiologique	FR	https://www.pollens.fr/	Permission to data re-use was requested and confirmed by the competent authority.
Nastavni Zadov Za Javno Zdravstvo	HR	https://zzjzpgz.hr/publikacije/	The right of access to information and the re-use of information are regulated and exercised in accordance with the Act on the Right of Access to Information (Official Gazette No. 25/13), which prescribes the principles of the right of access to information and re-use of information, restrictions on the right of access to information and re-use of information, procedure for exercising and protecting the right of access to information and re-use of information.<br>The right of access to information includes the right of the user to request and obtain information, as well as the obligation of public authorities to provide access to the requested information, i.e. to publish information independently of the request when such publication arises from an obligation determined by law or other regulation.<br>Re-use means the use of information by public authorities by natural or legal persons for commercial or non-commercial purposes other than the original purpose in the context of the public work for which that information was produced. The exchange of information between public authorities for the purpose of carrying out tasks falling within their scope shall not constitute re-use.
Medical University of Lodz	PL		The pollen data was provided on request by Prof. M. Chałubiński and Dr B. Majkowska-Wojciechowska from the Medical University of Lodz, Poland. DOI: 10.5114/aoms.2016.61978
Noise			
EEA: European Environment Agency	EU	https://www.eea.europa.eu/en	Unless otherwise indicated, the European Environment Agency (EEA) is the owner of copyrights and database rights in this website and its contents. The sources and owner(s) of the content are clearly indicated for each content.<br>Information, documents and material available on this website and for which the EEA holds the rights of use are public and may be re-used without prior permission, free of charge, for commercial or non-commercial purposes, provided that the EEA is always acknowledged as the original source of the material and that the original meaning or message of the content is not distorted.<br>Such acknowledgment must be included in each copy of the material. The re-use of the content on the EEA website covers the reproduction, adaptation and/or distribution, irrespective of the means and/or the format used. The re-use of certain data may be subject to different conditions, and if so the item concerned is accompanied by a copyright mark or other mention of the specific conditions relating to it.

Drinkable water			
ZWIK sp. z o.o., Zakładu Wodociągów i Kanalizacji sp. z o.o.	PL	https://zwik.lodz.pl/	Permission to data re-use was requested and confirmed by the competent authority.
Manchester Water Works, City of Manchester	UK	https://www.manchesternh.gov/	All contents of the City of Manchester NH Official Website are: Copyright 2023 City of Manchester and/or its suppliers. All rights reserved. Materials uploaded to a Communication Service may be subject to posted limitations on usage, reproduction and/or dissemination. You are responsible for adhering to such limitations if you download the materials.
AMAP S.p.A., Azienda Municipalizzata Acquedotto di Palermo	IT	https://www.amapspa.it/	Data are freely available on the website.
data.gouv.fr.	FR	https://www.data.gouv.fr/en/	As part of your missions, you may produce, collect or use data, software source codes or algorithms. These represent sources of value for your organization, but also for other structures. The improvement in quality, documentation, openness and circulation of these resources are intended to improve the quality of your activities and to promote the creation of new services.
Águas e Energia do Porto	PT	https://www.aguasdoporto.pt/	The regime for access to administrative and environmental information and the reuse of administrative documents was approved by Law no. 26/2016, of 22 August - LADA, and is applicable to all bodies that make up the Public Administration, including the bodies of regional, municipal, intermunicipal or metropolitan companies, as well as any other local companies or public municipal services.<br><br>According to the provisions of Article 5 of the LADA, "everyone, without the need to declare any interest, has the right of access to administrative documents, which includes the rights of consultation, of reproduction and of information about their existence and content", except for restrictions to the right of access under the provisions of Article 6 of the said diploma.
Aigües de Reus	ES	https://www.aiguesdereus.cat/es-es/El-Agua-en-Reus/Calidad-del-agua	The mere viewing, printing, downloading or temporary storage, either in whole or in part, of the contents and / or the elements inserted by AIGÜES DE REUS on the Web exclusively for personal, private and non-profit use by the User is authorized, provided that, in any case, the origin and / or author of the same is indicated and that, Where appropriate, the copyright symbol and/or industrial property notes of their owners appear. Any other use or exploitation of any rights will be subject to the prior and express authorization specifically granted for this purpose by AIGÜES DE REUS or the third party owner of the affected rights.
EYATH: Thessaloniki Water Supply & Sewerage Company SA.	GR	https://www.eyath.gr/	Permission to data re-use was requested and confirmed by the competent authority.
Nastavni Zadov Za Javno Zdravstvo	HR	https://zzjzpgz.hr/	The right of access to information and the re-use of information are regulated and exercised in accordance with the Act on the Right of Access to Information (Official Gazette No. 25/13), which prescribes the principles of the right of access to information and re-use of information, restrictions on the right of access to information and re-use of information, procedure for exercising and protecting the right of access to information and re-use of information.<br>The right of access to information includes the right of the user to request and obtain information, as well as the obligation of public authorities to provide access to the requested information, i.e. to publish information independently of the request when such publication arises from an obligation determined by law or other regulation.<br>Re-use means the use of information by public authorities by natural or legal persons for commercial or non-commercial purposes other than the original purpose in the context of the public work for which that information was produced. The exchange of information between public authorities for the purpose of carrying out tasks falling within their scope shall not constitute re-use.
VOKA d.o.o.: Vodovod Kanalizacija Celje, d.o.o.	SI	https://www.vo-ka-celje.si/sl/domov	The water data was provided on request by VOKA d.o.o.
Food Pesticides			
EFSA, European Food Safety Authority	EU	https://efsa.onlinelibrary.wiley.com/	Customers and their Authorized Users may download, search, retrieve, display and view, copy and save to a secure network or other electronic storage media and store or print out single copies of individual articles or items for their own personal use, scholarly, educational or scientific research or internal business use. Customers and Authorized Users may also transmit such material to a third-party colleague in hard copy or electronically for personal use or scholarly, educational, or scientific research or professional use but in no case for re-sale, systematic distribution, e.g. posting on a listserv, network (including scientific social networks) or automated delivery, or for any other use (including distribution through social networking websites and scholarly collaboration networks, except for those that have agreed to Wiley's Article Sharing Policy found here: https://authorservices.wiley.com/author-resources/Journal-Authors/Promotion/article-sharing-policy.html and solely in accordance therewith). In addition, Authorized Users have the right to use, with appropriate credit, figures, tables and brief excerpts from individual articles in the Licensed Electronic Product(s) in their own scientific, scholarly and educational works. Please note that these rights do not extend to the use of material, images or figures that are separately listed as the copyright of a third party.

Table S4. Data sources and copyright policies.

References – Supplementary Information

1 European Union, Copernicus Land Monitoring Service 2018, European Environment Agency (EEA)

2 QGIS Geographic Information System. Open Source Geospatial Foundation Project. http://qgis.osgeo.org

3 Parmes E, Pesce G, Sabel CE, Baldacci S, Bono R, Brescianini S, et al. Influence of residential land cover on childhood allergic and respiratory symptoms and diseases: Evidence from 9 European cohorts. *Environ Res* 2020;183:108953. (nr. 21 in the manuscript bibliography)

4 European Environment Agency. Air quality in Europe — 2020 report. EEA Report No 09/2020. Luxembourg: Publications Office of the European Union, 2020 (nr. 27 in the manuscript bibliography)

5 D'Amato G, Cecchi L, Bonini S, Nunes C, Annesi-Maesano I, Behrendt H, et al. Allergenic pollen and pollen allergy in Europe. *Allergy* 2007;62:976-90.

6 Cecchi L, D'Amato G, Annesi-Maesano I. External exposome and allergic respiratory and skin diseases. *J Allergy Clin Immunol* 2018;141:846-57. (nr. 2 in the manuscript bibliography)

7 ISPRA- Istituto Superiore per la Protezione e la Ricerca Ambientale. Stato e trend dei principali pollini allergenici in Italia (2003-2019). ISPRA 2021, Rapporti 338/2021, ISBN 978-88-448-1037-5

8 Directive (EU) 2020/2184 of the European Parliament and of the Council of 16 December 2020 on the quality of water intended for human consumption (recast) (nr. 34 in the manuscript bibliography)

9 Commission Directive 2009/90/EC laying down, pursuant to Directive 2000/60/EC of the European Parliament and of the Council, technical specifications for chemical analysis and monitoring of water status.

10 EFSA (European Food Safety Authority), Carrasco Cabrera L, Medina Pastor P, 2022. The 2020 European Union report on pesticide residues in food. *EFSA Journal* 2022;20:7215, 57 pp. https://efsa.onlinelibrary.wiley.com/doi/epdf/10.2903/j.efsa.2022.7215 (nr. 29 in the manuscript bibliography)

HEALS EXHES

Isabella Annesi-Maesano[16], Nour Baiz[16], Sandra Baldacci[1], Henrique Barros[18], John Bartzis[19], Norhidayah Binti Ahmad[20], Beatrice Bocca[21], Sonia Brescianini[21], Gemma Calamandrei[21], Anthoula Chatzimpaloglou[22], Eugenia Dogliotti[21], Ingrid Falnoga[14], Maria João Fonseca[18], Catherine Gabriel[3,4], Amir Gamil[16], Alberto Gotti[23], Wojciech Hanke[7], Milena Horvat[14], Joanna Jerzynska[8], Edward Johnstone[5], Joanna Jurewicz[7], Michael Kabesch[24], Katarzyna Kalska-Sochacka[7], Spyros Karakitsios[23], David Kocman[14], Vikas Kumar[25], Sara Maio[1], Darja Mazej[14], Filomena Mazzei[21], Ettore Meccia[21], Luisa Minghetti[21], Lorenza Nisticò[21], Eduardo de Oliveira Fernandes[26], Reena Perchard[5], Anna Pino[21], Kinga Polanska[7], Elisabete Ramos[9], Joaquim Rovira[10,11], Dimosthenis Sarigiannis[23], Marta Schumacher[25], Patrizia Silvi[1], Zdravko Spiric[15], Maria Antonietta Stazi[21], Kamila Szcześniak[7], Patrizia Tancredi[21], Janja Snoj Tratnik[14], Gabriela Ventura Silva[26], Giovanni Viegi[1]

[18] Institute of Public Health, University of Porto, Porto, Portugal

[19] University of Western Macedonia (UOWM) - Department of Mechanical Engineering, Sialvera and Bakola, Kozani, Greece

[20] Centre for Epidemiology, Division of Population Health, Health Services Research and Primary Care School of Health Sciences, Faculty of Biology, Medicine and Health, University of Manchester, Manchester, UK

[21] Italian National Institute of Health, Rome, Italy

[22] Aristotle University of Thessaloniki, Faculty of Chemistry, Thessaloniki, Greece

[23] Aristotle University of Thessaloniki, School of Engineering, Thessaloniki, Greece

[24] University Clinic Regensburg, Regensburg, Germany

[25] Environmental Engineering Laboratory, Universitat Rovira i Virgili, Tarragona, Spain

[26] Institute of Mechanical Engineering, University of Porto, Porto, Portugal

EarlyFOOD

Isabella Annesi-Maesano[16], Sandra Baldacci[1], Salvatore Fasola[17], Bart Keijser[27], Jasper Kieboom[27], Stefania La Grutta[17], Martin Larsen[28], Sara Maio[1], Velia Malizia[17], Laura Montalbano[17], Federico Pirona[1], Joaquim Rovira[10,11], Marta Schumacher[25], Patrizia Silvi[1], Ilaria Stanisci[1], Sofia Tagliaferro[1,2], Tim van den Broek[27], Rémy Villette[28].

[27] Netherlands Organisation for Applied Scientific Research (TNO), Leiden, The Netherlands

[28] Sorbonne Université, INSERM U1135, Centre d'Immunologie et des Maladies Infectieuses (CIMI-Paris), Paris, France
